# Predicting Outcome and Duration of Mechanical Ventilation in Acute Hypoxemic Respiratory Failure: The PREMIER Study

**DOI:** 10.3390/jcm14227903

**Published:** 2025-11-07

**Authors:** Jesús Villar, Jesús M. González-Martín, Cristina Fernández, Juan A. Soler, Marta Rey-Abalo, Juan M. Mora-Ordóñez, Ramón Ortiz-Díaz-Miguel, Lorena Fernández, Isabel Murcia, Denis Robaglia, José M. Añón, Carlos Ferrando, Dácil Parrilla, Ana M. Dominguez-Berrot, Pilar Cobeta, Domingo Martínez, Ana Amaro-Harpigny, David Andaluz-Ojeda, M. Mar Fernández, Estrella Gómez-Bentolila, Ewout W. Steyerberg, Luigi Camporota, Tamas Szakmany

**Affiliations:** 1 CIBER de Enfermedades Respiratorias, Instituto de Salud Carlos III, 28029 Madrid, Spain; josu.estadistica@gmail.com (J.M.G.-M.); jmaelizalde@gmail.com (J.M.A.); cafeoranestesia@gmail.com (C.F.); 2 Research Unit, Hospital Universitario Dr. Negrín, Fundación Canaria Instituto de Investigación Sanitaria, 35012 Las Palmas de Gran Canaria, Las Palmas, Spain; 3 Li Ka Shing Knowledge Institute, St. Michael’s Hospital, Toronto, ON M5B 1W8, Canada; 4 Faculty of Health Sciences, Universidad del Atlántico Medio, 35017 Tafira Baja, Las Palmas, Spain; 5 Research Unit, Hospital Universitario Dr. Negrín, 35019 Las Palmas de Gran Canaria, Spain; cristina.fersan76@gmail.com (C.F.); estrellagbentolila@gmail.com (E.G.-B.); 6 School and Post-doctoral Studies and Research, Universidad Fernando Pessoa Canarias, 35450 Guía, Las Palmas, Spain; 7 Intensive Care Unit, Hospital Universitario Virgen de la Arrixaca, 30120 El Palmar, Murcia, Spain; juasobar@hotmail.com (J.A.S.); dmbct1@gmail.com (D.M.); 8 Intensive Care Unit, Hospital Universitario A Coruña, 15006 La Coruña, Spain; marta.rey.abalo@sergas.es; 9 Intensive Care Unit, Hospital Universitario Regional Carlos Haya, 29010 Málaga, Spain; jumouci@gmail.com; 10 Intensive Care Unit, Hospital General Universitario de Ciudad Real, 13005 Ciudad Real, Spain; ramonortiz1980@hotmail.com; 11 Intensive Care Unit, Hospital Universitario Río Hortega, 47012 Valladolid, Spain; mlfernandezrod@saludcastillayleon.es; 12 Intensive Care Unit, Complejo Hospitalario Universitario de Albacete, 02006 Albacete, Spain; murciasaez.im@gmail.com; 13 Intensive Care Unit, Hospital Universitario Fundación Jiménez Díaz, 28040 Madrid, Spain; drobaglia@gmail.com; 14 Intensive Care Unit, Hospital Universitario La Paz, IdiPaz, 28029 Madrid, Spain; 15 Surgical Intensive Care Unit, Department of Anesthesia, Hospital Clinic, Institut d’Investigacions Biomèdiques August Pi i Sunyer (IDIBAPS), 08036 Barcelona, Spain; 16 Intensive Care Unit, Hospital Universitario N.S. de Candelaria, 38010 Santa Cruz de Tenerife, Spain; gudru74@yahoo.es; 17 Intensive Care Unit, Complejo Asistencial Universitario de León, 24008 León, Spain; adominguezb@saludcastillayleon.es; 18 Surgical Intensive Care Unit, Hospital Universitario Ramón y Cajal, 28034 Madrid, Spain; pilarcobeta@gmail.com; 19 Intensive Care Unit, Hospital Universitario Puerta de Hierro, 28222 Madrid, Spain; anaharpigny@gmail.com; 20 Intensive Care Unit, Complejo Asistencial Universitario de Palencia, 34005 Palencia, Spain; dandaluz@saludcastillayleon.es; 21 Intensive Care Unit, Hospital Universitario Mutua Terrassa, 08221 Terrassa, Barcelona, Spain; marufaes@yahoo.es; 22 Department of Biomedical Data Sciences, Leiden University Medical Center, 2333 ZG Leiden, The Netherlands; e.w.steyerberg@lumc.nl; 23 Julius Center, University Medical Center Utrecht, 3584 CX Utrecht, The Netherlands; 24 Department of Adult Critical Care, Guy’s and St Thomas’ NHS Foundation Trust, London SE1 9RT, UK; luigi.camporota@kcl.ac.uk; 25 Vice Chair for Research, Integrated Hospital Care Institute, Cleveland Clinic, Abu Dhabi 112412, United Arab Emirates

**Keywords:** acute hypoxemic respiratory failure, mechanical ventilation, lung-protective ventilation, multinomial regression, machine learning, prolonged mechanical ventilation, observational studies

## Abstract

**Objectives:** The ability of clinicians to predict prolonged mechanical ventilation (MV) in patients with acute hypoxemic respiratory failure (AHRF) is inaccurate, mainly because of the competitive risk of mortality. We aimed to assess the performance of machine learning (ML) models for the early prediction of prolonged MV in a large cohort of patients with AHRF. **Methods:** We analyzed 996 ventilated AHRF patients with complete data at 48 h after diagnosis of AHRF from 1241 patients enrolled in a prospective, national epidemiological study, after excluding 245 patients ventilated for <2 days. To account for competing mortality, we used multinomial regression analysis (MNR) to model prolonged MV in three categories: (i) ICU survivors (regardless of MV duration), (ii) non-survivors ventilated for 2–7 days, (iii) non-survivors ventilated for >7 days. We performed 4 × 10-fold cross-validation to validate the performance of potent ML techniques [Multilayer Perceptron (MLP), Support Vector Machine (SVM), Random Forest (RF)] for predicting patient assignment. **Results:** All-cause ICU mortality was 32.8% (327/996). We identified 12 key predictors at 48 h of AHRF diagnosis: age, specific comorbidities, sequential organ failure assessment score, tidal volume, PEEP, plateau pressure, PaO_2_, pH, and number of organ failures. MLP showed the best predictive performance [AUC 0.86 (95%CI: 0.80–0.92) and 0.87 (0.80–0.93)], followed by MNR [AUC 0.83 (0.76–0.90) and 0.84 (0.77–0.91)], in distinguishing ICU survivors, with non-survivors ventilated 2–7 days and >7 days, respectively. **Conclusions:** Accounting for ICU mortality, MLP and MNR offered accurate patient-level predictions. Further work should integrate clinical and organizational factors to improve timely management and optimize outcomes. This study was initially registered on 3 February 2025 at ClinicalTrials.gov (NCT06815523).

## 1. Introduction

Mechanical ventilation (MV) is essential for managing critically ill patients with acute hypoxemic respiratory failure (AHRF) in the intensive care unit (ICU). While most patients with severe AHRF require MV early in the course of their disease processes, prolonged MV may affect clinical decisions, including resource allocation, enteral feeding, or tracheostomy timing. Also, it is associated with complications, such as ventilator-associated pneumonia, ventilator-induced lung injury, sepsis, and multi-organ dysfunction, as well as healthcare costs and poorer outcomes [1,2,3]. The duration of MV is a major driver of treatment costs in critically ill patients [4].

Reducing the duration of MV is regarded as an important and actionable target goal in the management of AHRF patients. Accurate prediction of MV duration, particularly when prolonged, could support timely transfer to chronic or long-term healthcare facilities and enrollment into clinical trials [5]. However, early prediction of the duration of MV remains a major challenge for ICU clinicians [6,7] since there are important clinical considerations, such as defining goals of care, partnership with families, and burden of interventions. In practice, critical care physicians rely on their clinical judgment and heuristics to make predictions on the likely duration of MV, which are frequently inaccurate [6,7]. To date, no model has successfully predicted the duration of MV [8], particularly when MV is longer than 7 days, where outcomes are influenced by a complex interplay of institutional, ICU-level, and patient-associated factors. Advanced machine learning (ML) techniques may offer potential to improve prediction by reducing error margins [9,10]. While ML algorithms can extract patterns from large datasets, the overall performance of current ML models in predicting MV duration remains limited, despite the use of extensive clinical features and multiple modeling strategies [9,11,12].

Only a limited number of studies have successfully evaluated the role of ML in predicting the duration of prolonged MV in patients with AHRF [9,12,13,14]. The primary objective of this comprehensive analysis, termed the Prediction of Mechanical Ventilation in Acute Hypoxemic Respiratory Failure (PREMIER study), was to develop an ML model capable of predicting the duration of MV longer than 7 days early in the course of illness. We used data at 48 h after diagnosis of AHRF, while accounting for and separating the competing risk of early ICU mortality in patients admitted with AHRF of any etiology who required MV.

## 2. Methods

This study was approved by the Ethics Committee of Hospital Universitario Dr. Negrín, Las Palmas de Gran Canaria, Spain) (#CEI/CEIm 2024-383-1). Informed consent was waived in accordance with the Spanish legislation for biomedical research (Royal Decrees 1090/2015 and 957/2020) on the grounds that this study was a retrospective secondary analysis, there was anonymization/dissociation of data, and participation conferred neither risk nor benefit to patients (see Supplemental File). This study was conducted in compliance with the TRIPOD (transparent reporting of a multivariable prediction model for individual prognosis or diagnosis) reporting guidelines [15].

### 2.1. Patient Population and Study Design

We performed a comprehensive secondary analysis of an unrestricted, carefully harmonized dataset from our previously published PANDORA study [16]. The dataset comprised 1241 consecutive adult patients (≥18 years) with AHRF, defined by a PaO_2_/FiO_2_ ≤ 300 mmHg on positive end-expiratory pressure (PEEP) ≥5 cmH_2_O and FiO_2_ ≥ 0.3. Patients included had AHRF of any etiology and were managed with invasive mechanical ventilation using a lung-protective strategy across 22 ICUs in 14 geographical areas of Spain (Supplemental File).

Based on our previous work [9], we focused the current analysis on potentially clinically relevant variables collected at the time and day of AHRF diagnosis (T0), 24 h later (T24), and at 48 h (T48), always during or after ICU admission. Then, we analyzed patients with complete data from the first two full days of MV after AHRF diagnosis to evaluate the early probability of prolonged MV (defined as longer than 7 days from AHRF diagnosis), independent of the underlying disease or cause of death. We used representative data collected at 48 h after AHRF diagnosis. Patients who were extubated, discharged, or died before 48 h were excluded, as the model would have limited clinical applicability in such cases. No patients were excluded if they met all inclusion criteria, regardless of age, sex, underlying disease, estimated life expectancy, or duration of invasive MV. All included patients had arterial blood gases at enrollment, and SpO_2_ was not accepted as a surrogate for PaO_2_.

**Figure 1:**
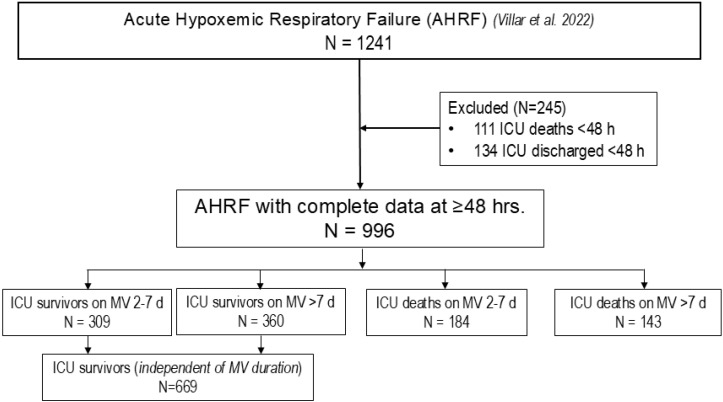
Flowchart of this study [16]. Abbreviations: AHRF: acute hypoxemic respiratory failure; d: days; h: hours; ICU: intensive care unit; MV: mechanical ventilation; N: number of patients.

Of 1241 patients (Table 1 and Table S1), 1015 patients were ventilated for more than 24 h (Table S2). We analyzed 996 patients (Table 1 and Table S3, Figure 1) after excluding 245 patients who received MV for <48 h after AHRF diagnosis (Table S4). This study was conducted in several steps. *In the first step*, we analyzed baseline data from the entire cohort of 1241 patients, and baseline data and at T48 of 996 patients on MV ≥2 days. Based on previous studies by our group [9], we selected risk features at 48 h of ICU management by forward–backward stepwise regression [17]. The forward selection approach starts with no variables and adds each new variable incrementally, testing for statistical significance, while the backward elimination method begins with a full model and then removes the least statistically significant variables one at a time. We decided against splitting by hospital since the largest hospital had, on average, fewer than 25 patients in each 2-month period of this study (Table S5). *In the second step*, patients were initially classified into four categories, based on ICU survival status and duration of MV (for 2 to 7 days or >7 days from AHRF diagnosis). As ICU survivors did not require readmission, all survivors were ultimately grouped together regardless of MV duration. This resulted in three groups: (i) ICU survivors, (ii) ICU non-survivors on MV for 2–7 days, and (iii) ICU non-survivors on MV > 7 days, irrespective of ICU discharge date. *In the third step*, we applied ML approaches to predict these outcome categories using selected variables.

Prolonged MV was defined as longer than 7 days after AHRF diagnosis, since the median of MV duration among 996 patients was 8 days (IQR 4–26 days). Because of the competitive risk of death in ventilated patients, ICU mortality could not be excluded from prediction models. Therefore, we applied a multinomial regression model using data collected at T48, given that most baseline variables and disease trajectories change substantially after ICU management [18]. We compared the performance of several ML methods and conventional statistics approaches in predicting three outcomes: ICU survival, ICU death on MV for 2–7 days, and ICU death on MV > 7 days after AHRF diagnosis (Figure 1).

### 2.2. Variables and Outcomes

The selection of potentially relevant variables was based on prior studies [9,19,20,21]. We collected demographics, comorbidities, cause of AHRF (or reason for MV), acute physiology and chronic health evaluation II (APACHE II) score [22] during the first 24 h of AHRF diagnosis, and data from ventilator settings and lung mechanics [tidal volume (VT), respiratory rate (RR), positive end-expiratory pressure (PEEP), plateau pressure (Pplat)], and gas exchange [(PaO_2_, PaCO_2_, FiO_2_, PaO_2_/FiO_2_ ratio, pH)] at T0, T24, and T48. We also recorded the sequential organ failure assessment (SOFA) score [23] and occurrence of extrapulmonary organ system failures (OFs) included in the SOFA scale (Supplemental File). We recorded the duration of MV, length of ICU and hospital stay, date and status (alive or dead) of patients at ICU and hospital discharge, and causes of death. Attending physicians recorded the causes of AHRF (or reasons for MV) and comorbidities (Supplemental File). We focused on variables collected at 48 h of MV after AHRF diagnosis to estimate the probability that a patient fit into one of the three predefined groups.

### 2.3. Predefined Rules and Statistical Analysis

Model selection criteria were defined a priori, prior to the conduct of the final statistical and ML analyses conducted (Supplemental File). Although in each patient, we recorded 246 variables per patient during their ICU stay, we analyzed the following as potential early predictors: age at ICU admission, sex, comorbidities, SOFA score, number of extrapulmonary OFs, PaO_2_, FiO_2_, PaO_2_/FiO_2_ ratio, PaCO_2_, pH, FiO_2_, VT, RR, PEEP, Pplat, driving pressure (calculated as the difference of Pplat minus PEEP), and minute ventilation (as an indirect measurement of dead space, calculated as VT x RR in liters/min) at 48 h after AHRF diagnosis. We did not include respiratory compliance in the model because it shares collinearity with three independent variables needed for its calculation (VT, Pplat, and PEEP), resulting in redundancy. Respiratory compliance did not contribute to predictive validity for mortality according to an expert panel in many cases of AHRF [24]. Although we have the APACHE II score of most patients, we excluded it as it is a composite score designed for the first 24 h of ICU admission (Supplemental File) and is less suitable for use at 48 h. Several variables shared collinearity with other independent variables (for example, calculation of PaO_2_/FiO_2_ ratio with FiO_2_ and PaO_2_ and Plat and PEEP for calculation of driving pressure). However, we considered all variables at the initial steps of analysis.

**Figure 2:**
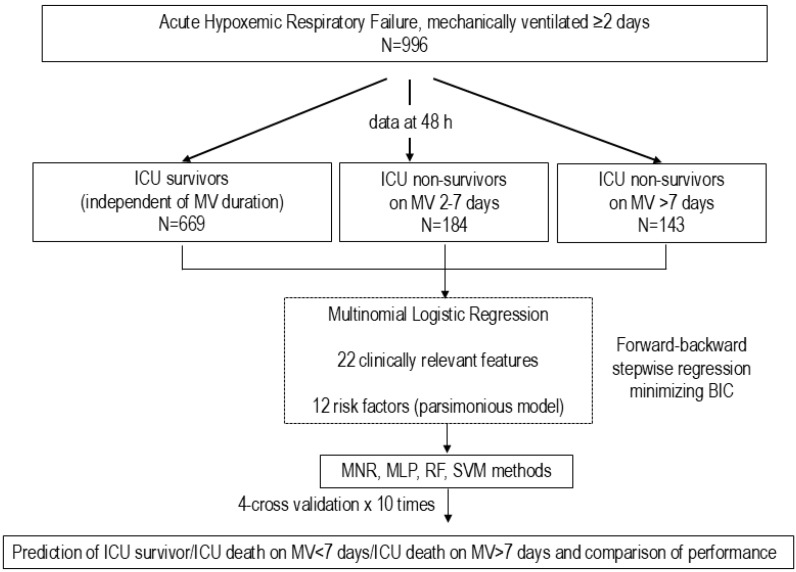
Diagram representing the study design of the PREMIER study. The diagram illustrates the scheme for the database of 996 patients with acute hypoxemic respiratory failure (AHRF) with data at 48 h from AHRF diagnosis, and the selection of clinically relevant variables for the final analysis. Once the most relevant variables were selected by forward–backward stepwise multinomial regression, we performed 4-fold cross-validation repeated 10 times using multinomial regression and three machine learning techniques and a comparison among the prediction models. Abbreviations: AHRF: acute hypoxemic respiratory failure; AUC: area under the receiver operating characteristic curve; BIC: Bayesian information criterion; MNR: multinominal regression; MLP: Multilayer Perceptron; MV: mechanical ventilation; RF: Random Forest; SVM: Support Vector Machine.

Since the inclusion of all available variables in ML can result in models that are overly complex and difficult to interpret, we aimed for parsimony by identifying a subset of features that allow sufficient performance while minimizing variables. We optimized the subset of selected variables by minimizing the Bayesian information criterion (BIC), using a multinomial regression model with three categories [25]. We performed statistical analysis using R (version 4.5.0. R Foundation for Statistical Computing, Vienna, Austria). A two-sided *p* < 0.005 was considered as evidence of prognostic strength [26]. We optimized the quality of models based on a 4-fold cross-validation approach repeated 10 times (Supplemental File). We assessed the best performance using multinomial regression analysis and three supervised ML algorithms (Multilayer Perceptron, Random Forest, and Support Vector Machine) [8,10,17,27]. Figure 1 and Figure 2, and Figure S1 summarize our analysis.

## 3. Results

A total of 245 AHRF patients were excluded (Table S4) because they had received MV for <48 h. A total of 996 patients, capturing more than 80% of the entire AHRF cohort, who received MV for ≥2 days were included (Table 1, Supplemental File). The most common reasons for MV in 996 patients with AHRF were stroke/coma, pneumonia, post-operative care, trauma, and sepsis. ICU mortality in the entire cohort (n = 1241) and the 996 cohort was similar [438/1241 (35%) vs. 327/996 (33%), respectively] (*p* = 0.22) (Tables S7 and S8), despite 111 patients dying in the ICU before 48 h of AHRF diagnosis. None of the baseline values from the selected relevant clinical features were statistically different between 1241 and 996 patients.

Of the total 996 patients analyzed, 669 patients (67%) were discharged alive from the ICU irrespective of MV duration: 309 were on MV for 2–7 days and 360 received MV > 7 days. A total of 51% (503/996) of patients received MV > 7 days of whom 360 survived the ICU and 143 died in the ICU. On average, at 48 h after AHRF diagnosis, ICU survivors had a lower severity of illness compared with those who subsequently died in the ICU (Table S9).

We studied 22 clinically relevant features (Table 2). Several variables were excluded for multicollinearity or for other reasons (Supplemental File). Using forward–backward stepwise regression, and minimizing the BIC error, a total of 12 variables collected at T48 after diagnosis of AHRF were selected as risk predictors (Table 2): age, specific comorbidities (arterial hypertension, diabetes, malignancy, chronic renal failure), SOFA score, VT, PEEP, Pplat, PaO_2_, pH, and number of extrapulmonary organ failures. The differences in coefficients, odds ratios, and statistical significance of the multinomial regression model for each variable in relation to the three ICU categories [ICU survivors, ICU deaths on MV for 2–7 days, and ICU deaths on MV >7 days] are reported in Table 3. Based on the OR (the highest value is associated with worsening), age, some comorbidities (arterial hypertension, diabetes, malignancy), SOFA, Pplat, PaO_2_, and the number of extrapulmonary organ failures were risk factors in the comparison of ICU survivors with deaths on MV for 2–7 days. Similarly, we found the same risk factors except chronic renal failure and PaO_2_ among ICU survivors with deaths on MV > 7 days (Table 3).

In the multinomial regression model, AUC was 0.85 (CI95% 0.82–0.882) and 0.87 (95%CI 0.84–0.90) in distinguishing ICU survivors from non-ICU survivors ventilated during ≤7 days and >7 days, respectively (Figure S2). However, in the machine learning process, MLP and MNR demonstrated the highest discriminatory performance according to AUC values (Table 4).

## 4. Discussion

Our findings highlight several strong risk factors for mortality or prolonged ventilation at 48 h after diagnosis of AHRF, including age, selected comorbidities, gas exchange, lung mechanics, and extrapulmonary organ dysfunction. These features reflect the pathophysiology of AHRF and might be modifiable by treatment [28,29]. While these individual factors are well established as risk markers in MV patients with acute respiratory failure, our study illustrates that it is the combination of variables that determines absolute risk. In practice, clinicians may not objectively account for the relationships with outcomes of the complex interactions between clinical variables, particularly in AHRF patients with diverse etiologies or indications for MV [28]. Our PREMIER model addresses this challenge by combining different events into a single outcome, recognizing that the duration of MV in AHRF reflects the patient’s health state and hence is closely related to survival. By predicting three distinct outcome categories—ICU survival (irrespective of MV duration), ICU death on MV for 2–7 days, and ICU death on MV >7 days—our approach provided meaningful prediction models [13,14]. Among the tested methods, MLP and MNR achieved the best accuracy for predicting allocation to the predefined clinical groups.

With respect to clinical features, our findings are consistent with the existing evidence: outcomes are worse with higher age [30]; certain comorbidities have a notable impact on survival in patients on MV [31]; patients with more severe lung damage had a lower PaO_2_/FiO_2_ ratio and require higher PEEP levels [32]; Pplat is directly associated with mortality [33,34]; multi-organ dysfunction increases the risk of death [18]; and acidemia is associated with worse neurological outcomes [35]. Although individual predictors are limited in discriminative ability, they may still serve as targets for modifiable interventions to shorten MV duration and improve outcomes. For instance, limiting Pplat may improve outcomes, while reducing the FiO_2_ to target lower oxygen saturations appears to be a safe intervention across a wide range of pathologies that result in AHRF [36]. Individualizing MV settings, including setting the PEEP using various tools, has a certain appeal; however, RCTs on the subject are equivocal [37]. Nevertheless, accurately predicting the course of MV in AHRF remains difficult due to competing mortality. Our model performed reasonably well in distinguishing mortality from survival, but only modestly in predicting prolonged MV as a primary outcome.

Patients with the same duration of MV could survive or die even under optimal ventilatory management [24,38]. Identifying those at higher risk of prolonged MV could directly influence important clinical decisions, including referral to specialized centers, or long-term ventilator units, timing of performing a tracheostomy [12,39], or alignment with goals of care [6]. In our patient population, fewer patients died in the ICU on MV > 7 days than patients on MV for 2–7 days [143/503 (28.4%) vs. 184/493 (37.3%), *p* = 0.003] (Table S8). Data show that patients who would benefit the most from modifiable timely therapeutic interventions to avoid adverse events [28] are those with the highest risk of death and prolonged MV duration that we identified in our study. Our findings suggest that outcomes can be predicted accurately as early as T48, although external validation in a large population of AHRF patients managed with lung-protective ventilation is needed. Prior evidence indicates that clinical judgment—despite relying on many of the same features used in our model—has poor accuracy in predicting prolonged MV [40]. Data-driven modeling may offer incremental improvements, but validation studies frequently report poor performance [41], mainly due to differences in data distributions, case-mix, timeframes, or outcome definitions [4,7,12,42]. Patterns of use of MV diverge across countries [43] and could reflect differences among patients, clinician preferences, and the organization of healthcare systems. For example, permissive hypercapnia is occasionally required to allow lung-protective MV, and pH could transiently decrease but gradually improves in 72 h following metabolic compensation. While this strategy may help reduce VT and improve oxygenation in some patients, it also carries a risk of cardiovascular compromise and organ dysfunction [44]. Similar interdependencies exist among other predictors in our model.

Our study has several strengths. First, we included patients from a multicenter network representing current clinical practice in AHRF. Second, since we developed a model that considers death in the prediction of MV duration, our work could accelerate clinical research in the field. Third, our study is different from others in that we considered a mix of different outcomes in the prediction of MV duration, such as death. Fourth, our cohort captured more than 80% of the entire AHRF population, which supports representativeness. Fifth, we based the modeling framework on established clinical knowledge, enhancing interpretability and potential clinical utility. However, several limitations must be acknowledged. First, we did not stratify predictions by the underlying etiologies of AHRF, although the broad inclusion criteria and sample size may mitigate this concern. Second, we did not capture detailed data on factors influencing weaning, such as fluid balance, sedation, neuromuscular blockade, secondary infections, or muscle strength. Third, our dataset predates the COVID-19 pandemic, during which ventilatory practices may have varied [45]. Fourth, we trained our algorithms using three ML techniques; other ML techniques might lead to better prediction models. Fifth, we did not evaluate the wider context of decision making for the discontinuation of MV. Recent evidence suggests that several different weaning strategies can influence outcomes [46,47].

## 5. Conclusions

Accounting for ICU mortality, MLP and MNR offered accurate patient-level predictions in AHRF patients. Further work should integrate clinical and organizational factors to improve timely management and optimize outcomes.

## Figures and Tables

**Table 1:** Baseline characteristics and outcome data of 1241 ventilated patients with acute hypoxemic respiratory failure (AHRF) and 996 patients with data at 48 h after AHRF diagnosis.

Variables	N = 1241 T0	N = 996 T0	*p*-Value
Age, years, median (IQR) Age, years, mean ± SD	65 (54–74) 62.8 ± 14.3	65 (54–73) 62.1 ± 14.6	0.254 0.254
Sex	n (%: 95%CI)	N (%: 95%CI)	
Male	834 (67.2: 64.6 to 69.8)	680 (68.3: 65.4 to 71.2)	0.580
Female	407 (32.8: 30.2 to 35.4)	316 (31.7; 28,8 to 34.6)	0.580
Etiology (reasons for invasive MV), n (%: 95%CI)			
Post-surgery	208 (16.8: 14.7 to 18.8)	144 (14.5: 12.3 to 16.6)	0.138
Stroke or coma	191 (15.4: 13.4 to 17.4)	162 (16.3: 14.0 to 18.6)	0.562
Pneumonia	169 (13.6: 11.7 to 15.5)	149 (15.0: 12.7 to 17.2)	0.346
Sepsis/acute pancreatitis	152 (12.3;10.4 to 14.1)	118 (11.8: 9.8 to 13.9)	0.718
Trauma	151 (12.2: 10.4 to 14.0)	135 (13.6: 11.4 to 15.7)	0.325
Cardiac arrest	117 (9.4: 7.8 to 11.1)	88 (8.8: 7.1 to 10.6)	0.625
Cardiac failure/fluid overload	62 (5.0: 3.8 to 6.2)	49 (4.9: 3.6 to 6.3)	0.914
Aspiration/inhalation	49 (4.0: 2.9 to 5.0)	45 (4.5: 3.2 to 5.8)	0.559
Others	137 (11.0: 9.3 to 12.8)	101 (10.1: 8.3 to 12.0)	0.492
Unknown etiology	5 (0.4: 0 to 0.7)	5 (0.5: 0.1 to 0.9)	0.724
APACHE II score, mean ± SD	21.0 ± 8.0 ^§^	20.7 ± 7.5	0.365
SOFA score, mean ± SD	8.95 ± 3.47	8.93 ± 3.27	0.890
FiO_2_, mean ± SD	0.63 ± 0.22	0.64 ± 0.22	0.285
PaO_2_, mmHg, mean ± SD	98.9 ± 34.6	98.8 ± 34.7	0.946
PaO_2_/FiO_2_, mmHg, mean ± SD	170.5 ± 64.1	169.9 ± 64.7	0.827
PaCO_2_, mmHg, mean ± SD	46.1 ±12.4	46.1 ± 12.1	1.0
pH, mean ± SD	7.32 ± 0.11	7.32 ± 0.11	1.0
VT, mL/kg PBW, mean ± SD	6.88 ± 1.07	6.90 ± 1.06	0.659
Respiratory rate, ventilator cycles/min, mean ± SD	19.7 ± 4.4	19.9 ± 4.4	0.285
Minute ventilation, L/min, mean ± SD	8.6 ± 2.1	8,7 ± 2.1	0.263
PEEP, cmH_2_O, mean ± SD	7.8 ± 2.8	8.0 ± 2.9	0.100
Plateau pressure, cmH_2_O, mean ± SD	22.3 ± 5.5	22.3 ± 5.4	1.0
Driving pressure, cmH_2_O, mean ± SD	14.5 ± 4.9	14.3 ± 4.7	0.329
No. extrapulmonary OF, mean ± SD	1.72 ± 1.05	1.70 ± 1.01	0.649
Days from last day of MV to ICU discharge, median (IQR)	2 (0–5)	2 (0–6)	0.247
All-cause ICU mortality, n (%: 95%CI)	438 (35.3: 32.6 to 38.0)	327 (32.8: 29.9 to 35.8)	0.216
All-cause hospital mortality, n (%: 95%CI)	514 (41.4: 38.7 to 44.2)	393 (39.5: 36.4 to 42.5)	0.363

APACHE: acute physiology and chronic health evaluation; AHRF: acute hypoxemic respiratory failure; CI: confidence intervals; d: days; FiO_2_: fraction of inspired oxygen concentration; ICU: intensive care unit; IQR: interquartile range; MV: mechanical ventilation; OF: organ failure; PBW: predicted body weight; PEEP: positive end-expiratory pressure; SD: standard deviation; SOFA: sequential organ failure assessment scale; T0: at AHRF diagnosis; VT: tidal volume. ^§^ APACHE II was not reported at baseline in 40 patients from the entire 1241 cohort, and in 35 from the 996 patients.

**Table 2:** Selection of clinically relevant variables collected in 996 patients with acute hypoxemic respiratory failure (AHRF) at 48 h after AHRF diagnosis by forward–backward stepwise regression using a forward–backward multinomial regression model minimizing BIC (ICU survivors, ICU non-survivors ventilated ≤7 days, and ICU non-survivors ventilated >7 days).

Variables at T48	Selected Variables
Age	X
Sex	
Arterial hypertension (comorbidity)	X
Diabetes (comorbidity)	X
Obesity (comorbidity)	
COPD (comorbidity)	
Cardiac failure (comorbidity)	
Malignancy (comorbidity)	X
Immunocompromised (comorbidity)	
Chronic renal failure (comorbidity)	X
SOFA score	X
VT (kg/min PBW)	X
FiO_2_	
Respiratory rate	
PEEP	X
Plateau pressure	X
PaO_2_	X
PaO_2_/FiO_2_ ratio	
PaCO_2_	
pH	X
Number of organ failures	X
Minute ventilation (liters/min)	

Abbreviations: COPD: chronic obstructive pulmonary disease; FiO_2_: fraction of inspiratory oxygen; PBW: predicted body weight; PEEP: positive end-inspiratory pressure; SOFA: sequential organ failure assessment score; VT: tidal volume.

**Table 3:** Coefficients, odds ratio, and statistical significance of the multinomial regression model for each variable (n = 12) in relation to the three categories [ICU survivors (independent of duration of mechanical ventilation), ICU death on MV ≤7 days, and ICU deaths on MV > 7 days] in 996 patients with acute hypoxemic respiratory failure (AHRF) ventilated for ≥2 days.

Variables	ICU Survivors vs. ICU Deaths on MV ≤ 7 Days				ICU Survivors vs. ICU Deaths on MV > 7 Days			
	β	SE	OR (95% CI)	*p*-Value	β	SE	OR (95% CI)	*p*-Value
Intercept	35.6	0.02	2.78 × 10^15^ (2.68–2.89)	<0.001	1.47	0.02	4.33 (4.16–4.50)	<0.001
Age	0.03	0.01	1.03 (1.02–1.05)	<0.001	0.04	0.01	1.04 (1.02–1.06)	<0.001
Arterial hypertension: No	0 (ref)	-	1 (ref)	-	0 (ref)	-	1 (ref)	-
Arterial hypertension: Yes	0.5	0.23	1.65 (1.04–2.62)	0.032	0.05	0.25	1.05 (0.64–1.71)	0.856
Diabetes: No	0 (ref)	-	1 (ref)	-	0 (ref)	-	1 (ref)	-
Diabetes: Yes	0.04	0.23	1.04 (0.66–1.65)	0.853	0.63	0.24	1.88 (1.17–3.03)	0.009
Malignancy: No	0 (ref)	-	1 (ref)	-	0 (ref)	-	1 (ref)	-
Malignancy: Yes	0.01	0.30	1.01 (0.56–1.81)	0.969	0.56	0.29	1.74 (0.99–3.08)	0.056
Chronic renal failure: No	0 (ref)	-	1 (ref)	-	0 (ref)	-	1 (ref)	-
Chronic renal failure: Yes	−0.7	0.38	0.49 (0.24–1.04)	0.062	0.10	0.35	1.10 (0.55–2.21)	0.783
SOFA T48	0.1	0.06	1.14 (1.02–1.28)	0.026	0.01	0.06	1.01 (0.89–1.15)	0.846
VT (kg/min/PBW) T48	−0.2	0.10	0.78 (0.64–0.96)	0.019	−0.39	0.11	0.68 (0.55–0.83)	<0.001
PEEP T48	−0.3	0.04	0.73 (0.67–0.80)	<0.001	−0.24	0.04	0.79 (0.72–0.86)	<0.001
Pplat T48	0.2	0.02	1.24 (1.19–1.30)	<0.001	0.26	0.02	1.30 (1.23–1.36)	<0.001
PaO_2_ T48	0.01	0	1.01 (1–1.01)	0.019	0	0	1 (1–1.01)	0.293
pH T48	−5.7	0.14	0 (0–0)	<0.001	−1.10	0.15	0.33 (0.25–0.45)	<0.001
Number of organ failures T48	0.6	0.19	1.73 (1.18–2.52)	0.005	0.73	0.21	2.08 (1.38–3.13)	<0.001

Abbreviations: MV: mechanical ventilation; OR: Odds ratio; PEEP: positive end-expiratory pressure; Pplat: plateau pressure; SE: standard error; SOFA: sequential organ failure assessment; VT: tidal volume; T48: at 48 h after diagnosis of AHRF.

**Table 4:** Comparison of analysis of early prediction of performance using Multilayer Perceptron, Random Forest, Support Vector Machine, and Multinomial Regression of ICU survivors (independent of duration of mechanical ventilation) and ICU deaths on MV ≤7 days or ICU deaths on MV > 7 days in 966 patients with acute hypoxemic respiratory failure with complete data at 48 h. AUC is a measure of discriminative ability. Global is for the 3 categories.

Techniques	Global AUC (95%CI)	Accuracy AUC (95% CI)	ICU Survivors vs. ICU Deaths on MV ≤ 7 Days AUC (95% CI)	ICU Survivors vs. ICU Deaths on MV > 7 Days AUC (95% CI)
Multilayer Perceptron	0.78 (0.74–0.81)	0.74 (0.68–0.78)	0.86 (0.80–0.92)	0.86 (0.80–0.93)
Random Forest	0.73 (0.70–0.76)	0.70 (0.65–0.75)	0.79 (0.72–0.87)	0.78 (0.69–0.86)
Support Vector Machine	0.66 (0.60–0.69)	0.67 (0.63–0.71)	0.66 (0.0.58–0.75)	0.73 (0.64–0.82)
Multinomial Regression	0.75 (0.73–0.78)	0.72 (0.67–0.76)	0.83 (0.76–0.90)	0.84 (0.77–0.91)

Abbreviations: AUC: area under the receiver operating curve; CI: confidence interval; ICU: intensive care unit; MV: mechanical ventilation.

## Data Availability

All data needed to evaluate the conclusions in this article are presented and tabulated in the main text or the Supplemental File. Data are available from the corresponding author on reasonable request.

## Supplementary Materials

The following supporting information can be downloaded at https://www.mdpi.com/article/10.3390/jcm14227903/s1.

## References

[1] Rosenthal V.D., Memish Z.A., Bearman G. (2025). Preventing ventilator-associated pneumonia: A position paper of the International Society for Infectious Diseases, 2024 update. Int. J. Infect. Dis..

[2] Slutsky A.S., Ranieri V.M. (2013). Ventilator-induced lung injury. N. Engl. J. Med..

[3] Singer M., Deutschman C.S., Seymour C.W., Shankar-Hari M., Annane D., Bauer M., Bellomo R., Bernard G.R., Chiche J.D., Coopersmith C.M. (2016). The third international consensus definitions for sepsis and septic shock (sepsis-3). JAMA.

[4] Dasta J.F., McLaughlin T.P., Mody S.H., Piech C.T. (2005). Daily cost of an intensive care unit day: The contribution of mechanical ventilation. Crit. Care Med..

[5] Slutsky A.S., Villar J. (2019). Early paralytic agents for ARDS? Yes, no, and sometimes. N. Engl. J. Med..

[6] Figueroa-Casas J.B., Connery S.M., Montoya R., Dwivedi A.K., Lee S. (2014). Accuracy of early prediction of duration of mechanical ventilation by intensivists. Ann. Am. Thorac. Soc..

[7] Troché G., Moine P. (1997). Is the duration of mechanical ventilation predictable?. Chest.

[8] Xu Y., Xue J., Deng Y., Tu L., Ding Y., Zhang Y., Yuan X., Xu K., Guo L., Gao N. (2025). Advances in machine learning for mechanically ventilated patients. Int. J. Gen. Med..

[9] Villar J., González-Martín J.M., Fernández C., Soler J.A., Ambrós A., Pita-García L., Fernández L., Ferrando C., Arocas B., González-Vaquero M. (2024). Predicting the length of mechanical ventilation in acute respiratory disease syndrome using machine learning: The PIONEER study. J. Clin. Med..

[10] Mendiratta S., Mukkelli V.G., Kayal E.B., Khanna P., Mehndiratta A. (2025). Predicting mechanical ventilation duration I ICU patients: A data-driven machine learning approach for clinical decision-making. Digit. Health.

[11] Dam T.A., Roggeveen L.F., van Diggelen F., Fleuren L.M., Jagesar A.R., Otten M., de Vries H.J., Gommers D., Cremer O.L., Bosman R.J. (2022). Predicting responders to prone positioning in mechanically ventilated patients with COVID-19 using machine learning. Ann. Intensive Care.

[12] Parreco J., Hidalgo A., Parks J.J., Kozol R., Rattan R. (2018). Using artificial intelligence to predict prolonged mechanical ventilation and tracheostomy placement. J. Surg. Res..

[13] Ding X.F., Li J.B., Liang H.Y., Wang Z.Y., Jiao T.T., Liu Z., Yi L., Bian W.S., Wang S.P., Zhu X. (2019). Predictive model for acute respiratory distress syndrome events in ICU patients in China using machine learning algorithms: A secondary analysis of a cohort study. J. Transl. Med..

[14] Wang Z., Zhang L., Huang T., Yang R., Cheng H., Wang H., Yin H., Lyu J. (2023). Developing an explainable machine learning model to predict the mechanical ventilation duration of patients with ARDS in intensive care units. Heart Lung.

[15] Collins G.S., Reitsma J.B., Altman D.G., Moons K.G. (2015). Transparent reporting of a multivariable prediction model for individual prognosis or diagnosis (TRIPOD): The TRIPOD statement. BMJ.

[16] Villar J., Mora-Ordoñez J.M., Soler J.A., Mosteiro F., Vidal A., Ambrós A., Fernández L., Murcia I., Civantos B., Romera M.A. (2022). The PANDORA study: Prevalence and outcome of acute hypoxemic respiratory failure in the pre-Covid-19 era. Crit. Care Explor..

[17] Calster B.V., Vergouwe Y., Looman C.W.N., Belle V.V., Timmerman D., Steyerberg E.W. (2012). Assessing the discriminative ability of risk models for more than two outcome categories. Eur. J. Epidemiol..

[18] Villar J., Martínez D., Mosteiro F., Ambrós A., Añón J.M., Ferrando C., Soler J.A., Montiel R., Vidal A., Conesa-Cayuela L.A. (2018). Is overall mortality the right composite endpoint in clinical trials of acute respiratory distress syndrome?. Crit. Care Med..

[19] Villar J., Ambrós A., Mosteiro F., Martínez D., Fernández L., Ferrando C., Carriedo D., Soler J.A., Parrilla D., Hernández M. (2019). A prognostic enrichment strategy for selection of patients with acute respiratory distress syndrome in clinical trials. Crit. Care Med..

[20] Dai Q., Wang S., Liu R., Wang H., Zheng J., Yu K. (2019). Risk factors for outcomes of acute respiratory distress syndrome patients: A retrospective study. J. Thorac. Dis..

[21] Le S., Pellegrini E., Green-Saxena A., Summers C., Hoffman J., Calvert J., Das R. (2020). Supervised machine learning for the early prediction of acute respiratory distress syndrome (ARDS). J. Crit. Care.

[22] Knaus W.A., Draper E.A., Wagner D.P., Zimmerman J.E. (1985). APACHE II: A severity of disease classification system. Crit. Care Med..

[23] Vincent J.L., de Mendonça A., Cantraine F., Moreno R., Takala J., Suter P.M., Sprung C.L., Colardyn F., Blecher S. (1998). Use of the SOFA score to assess the incidence of organ dysfunction/failure in Intensive Care Units: Results of a multicenter, prospective study. Working group on “sepsis-related problems” of the European Society of Intensive Care Medicine. Crit. Care Med..

[24] Acute Respiratory Distress Syndrome Network (2000). Ventilation with lower tidal volumes as compared with traditional tidal volumes for acute lung injury and the acute respiratory distress syndrome. N. Engl. J. Med..

[25] Vrieze S.I. (2012). Model selection and psychological theory: A discussion of the differences between the Akaike information criterion (AIC) and the Bayesian information criterion (BIC). Psychol. Methods.

[26] Ioannidis J.P.A. (2018). The proposal to lower p value thresholds to 0.005. JAMA.

[27] Rashid M., Ramakrishnan M., Chandran V.P., Nandish S., Nair S., Shanbhag V., Thunga G. (2022). Artificial intelligence in acute respiratory distress syndrome: A systematic review. Artif. Intell. Med..

[28] Maslove D.M., Tang B., Shankar-Hari M., Lawler P.R., Angus D.C., Baillie J.K., Baron R.M., Bauer M., Buchmann T.G., Calfee C.S. (2022). Redefining critical illness. Nat. Med..

[29] Smit M.R., Reddy K., Munshi L., Bos L.D.J. (2025). Towards precision medicine in respiratory failure. Crit. Care Med..

[30] Gee M.H., Gotlieb J.E., Albertine K.H., Kubis J.M., Peters S.P., Fish J.E. (1990). Physiology of aging related to outcome in the adult respiratory distress syndrome. J. Appl. Physiol..

[31] Heybati K., Deng J., Bhandarkar A., Zhou F., Zamanian C., Arya N., Bydon M., Bauer P.R., Gajic O., Walkey A.J. (2024). Outcomes of acute respiratory failure in patients with cancer in the United States. Mayo Clin. Proc..

[32] Villar J., Fernández C., González-Martín J.M., Ferrando C., Añón J.M., Del Saz-Ortíz A.M., Díaz-Lamas A., Bueno-González A., Fernández L., Domínguez-Berrot A.M. (2022). Respiratory subsets in patients with moderate to severe acute respiratory distress syndrome for early prediction of death. J. Clin. Med..

[33] Shiu K.K., Rosen M.J. (2006). Is there a safe plateau pressure threshold for patients with acute lung injury and acute respiratory distress syndrome?. Am. J. Respir. Crit. Care Med..

[34] Ball L., Battaglini D., Pelosi P. (2016). Postoperative respiratory disorders. Curr. Opin. Crit. Care.

[35] Yoshida R., Komukai K., Kubota T., Kinoshita K., Fukushima K., Yamamoto H., Niijima A., Matsumoto T., Nakayama R., Watanabe M. (2024). The relationship between the initial pH and neurological outcome in patients with out-of-hospital cardiac arrest is affected by the status of recovery of spontaneous circulation on hospital arrival. Heart Vessels.

[36] Martin D.S., Gould D.W., Shahid T., Doidge J.C., Cowden A., Sadique Z., Camsooksai J., Charles W.N., Davey M., Francis-Johnson A. (2025). Conservative oxygen therapy in mechanically ventilated critically ill patients: The UK-ROX randomized clinical trial. JAMA.

[37] Yuan X., Chen D., Chao Y., Zhang R., Guo L., Xie J., Liu S., Zhao Z., Huang Y., Yang Y. (2025). Effect of individualized positive end-expiratory pressure titrated by electrical impedance tomography in patients with acute respiratory distress syndrome. Am. J. Respir. Crit. Care Med..

[38] Churpek M.M., Gupta S., Spicer A.B., Parker W.F., Fahrenbach J., Brenner S.K., Leaf D.E., for the STOP-COVID investigators (2021). Hospital-level variation in death for critically ill patients with COVID-19. Am. J. Respir. Crit. Care Med..

[39] Szakmany T., Russell P., Wilkes A.R., Hall J.E. (2015). Effect of early tracheostomy on resource utilization and clinical outcomes in critically ill patients: Meta-Analysis of randomized controlled trials. Brit. J. Anaesth..

[40] Young D., Harison D.A., Cuthbertson B.H., Rowan K., TracMan Collaborators (2013). Effect of early vs late tracheostomy placement on survival in patients receiving mechanical ventilation: The TracMan randomized trial. JAMA.

[41] Retel Helmrich I.R.A., Mikolić A., Kent D.M., Lingsm H.F., Wynants L., Steyerberg E.W., van Klaveren D. (2022). Does poor methodological quality of prediction modeling studies translate to poor model performance? An illustration in traumatic brain injury. Diagn. Progn. Res..

[42] Rose L., McGinlay M., Amin R., Burns K.E., Connolly B., Hart N., Jouvet P., Katz S., Leasa D., Mawdsley C. (2017). Variation in definition of prolonged mechanical ventilation. Respir. Care.

[43] Jivraj N.K., Hill A.D., Shiah M.S., Hua M., Gershengorn H.B., Ferrando-Vivas P., Harrison D., Rowan K., Lindenauer P.K., Wunsch H. (2023). Use of mechanical ventilation across 3 countries. JAMA Intern. Med..

[44] Romano T.G., Correia M.D.T., Mendes P.V., Zampieri F.G., Maciel A.T., Park M. (2016). Metabolic acid-base adaptation triggered by acute persistent hypercapnia in mechanically ventilated patients with acute respiratory distress syndrome. Rev. Bras. Ter. Intensiva.

[45] Grasselli G., Calfee C.S., Camporota L., Poole D., Amato M.B.P., Antonelli M., Arabi Y.M., Baroncelli F., Beitler J.R., Bellani G. (2023). ESICM guidelines on acute respiratory distress syndrome: Definition, phenotyping and respiratory support strategies. Intensive Care Med..

[46] Perkins G.D., Mistay D., Gates S., Gao F., Suelson C., Hart N., Camporota L., Karley J., Carle C., Paramasivana E. (2018). Effect of protocolized weaning with early extubation to noninvasive ventilation vs. invasive weaning on time to liberation from mechanical ventilation among patients with respiratory failure. The Breathe randomized clinical trial. JAMA.

[47] Burns K.E.A., Rizvi L., Cook D.J., Lebovic G., Dodek P., Villar J., Slutsky A.S., Jones A., Kapadia F.N., Gattas D.J. (2021). Ventilator weaning and discontinuation practice for critically ill patients. JAMA.

