# Supplementary material for: Predicting Outcome and Duration of Mechanical Ventilation in Acute Hypoxemic Respiratory Failure: The PREMIER Study
Source: J Clin Med. 2025 Nov 7;14(22):7903. doi: 10.3390/jcm14227903 (PMC12653734; doi:10.3390/jcm14227903)
Supplement: Supplementary file 1 [file jcm-14-07903-s001.zip › jcm-3930758-supplementary.pdf]

SUPPLEMENTAL FILE

This Supplemental File has been provided by the authors for additional information about their work.

Predicting outcome and duration of mechanical ventilation in Acute Hypoxemic Respiratory Failure: The PREMIER Study

Jesús Villar, Jesús M. González-Martín, Cristina Fernández, Juan A. Soler,
Marta Rey-Abalo, Juan M. Mora-Ordoñez, Ramón Ortiz-Díaz-Miguel, Lorena Fernández,
Isabel Murcia, Denis Robaglia, José M. Añón, Carlos Ferrando, Dácil Parrilla,
Ana M. Domínguez-Berrot, Pilar Cobeta, Domingo Martínez, A. Amaro-Harpigny,
D. Andaluz-Ojeda, M. Mar Fernández, Estrella Gómez-Bentolila,
Ewout W. Steyerberg, Luigi Camporota, Tamas Szakmany

For the **PREdiction of MechanIcal vEntilation in acute hypoxemic Respiratory failure** (PREMIER) network

Corresponding author:
Jesús Villar, MD, PhD
Research Unit at Hospital Universitario Dr. Negrín, Fundación Canaria Instituto de Investigación Sanitaria de Canarias, Las Palmas de Gran Canaria, Spain.
Barranco de la Ballena s/n, Annex building. 35019 Las Palmas de Gran Canaria, Spain.
Email: jesus.villar54@gmail.com

The PREMIER study – Supplementary Methods

TABLE OF CONTENTS

Abbreviations	page 3
**SUPPLEMENTARY METHODS**	page 4
Ethics approval	page 4
Patient population	page 4
Study design	page 5
General care	page 7
Data collection and follow-up	page 8
Statistical analysis plan	page 10
**SUPPLEMENTARY RESULTS**	page 13
**SUPPLEMENTARY DISCUSSION**	page 14
Tables S1 to S10	page 14-28
Figures S1 – S3	page 29-31
**SUPPLEMENTARY REFERENCES**	page 32
**APPENDIX S1**	
List of centers and investigators involved in the PREMIER study	page 36-38

ABBREVIATIONS

**AHRF**	Acute hypoxemic respiratory failure
**APACHE**	Acute physiology and chronic health evaluation score
**AUC ROC**	area under the receiver operating characteristic curve
**BIC**	Bayesian information criterion
**CI**	Confidence interval
**COPD**	Chronic obstructive pulmonary disease
**FiO_2**	Fraction of inspired oxygen
**ICU**	Intensive care unit
**IQR**	Interquartile range
**ML**	Machine learning
**MLP**	Multilayer Perceptron
**MNR**	Multinomial regression
**MV**	Mechanical ventilation (invasive)
**NIV**	Non-invasive ventilation
**OF**	Organ failure
**OR**	Odds ratio
**PREMIER**	PREdiction of MechanIcal vEntilation in acute hypoxemic Respiratory failure
**PaO_2**	Partial pressure of oxygen in arterial blood
**PaO_2/FiO_2**	Partial pressure of oxygen in arterial blood to fraction of inspired oxygen ratio
**PBW**	Predicted body weight
**PEEP**	Positive end-expiratory pressure
**Pplat**	Plateau pressure
**RCT**	Randomized controlled trial
**RF**	Random Forest
**RL**	Logistic Regression
**RM**	Recruitment maneuver
**RR**	Risk ratio
**SD**	Standard deviation
**SE**	Standard error
**SOFA**	Sequential organ failure assessment score
**SpO2**	Peripheral oxygen saturation
**SVM**	Support Vector Machine
**VFD**	Ventilator-free days
**VT**	Tidal volume
**T0**	At the time of diagnosis of AHRF
**T24**	24 h after diagnosis of AHRF
**T48**	48 H after diagnosis of AHRF

SUPPLEMENTARY METHODS

This comprehensive analysis, termed the **Prediction of Mechanical Ventilation in Acute Hypoxemic Respiratory Failure** (PREMIER Study), was an investigator-initiated clinical study from a multicenter, non-interventional, observational study, conducted in a network of intensive care units (ICUs) from several geographical areas of Spain (Appendix 1). The PREMIER study was initially registered at ClinicalTrial.gov on February 3, 2025 (NCT06815523).

Ethics Approval

This study was approved by the Ethics Committees of Hospital Universitario Dr. Negrín (Las Palmas de Gran Canaria, Spain) (#CEI/CEIm 2024-383-1). Requirement for informed consent was waived under the Royal Decrees 1090/2015 and 957/2020 based on the Spanish legislation for biomedical research due to the retrospective nature of this secondary analysis, the anonymization and dissociation of data, and no harm and no benefit for managing patients.

We used an unrestricted dataset from our previously published PANDORA study in patients with acute hypoxemic respiratory failure (AHRF) [1], after being approved by the Ethics Committees of Hospital Universitario La Paz, Madrid, Spain (#PI-2694), and Hospital Clínico Universitario de Valladolid, Spain (#PI17-594), and adopted by all participating centers. This study was conducted in accordance with the fundamental principles established in the Declaration of Helsinki, the Convention of the European Council related to human rights and biomedicine, the Ethical Guidelines for Health-related Research Involving Humans by the Council for International Organization of Medical Sciences of the World Medical Association [2], and within the requirements established by the Spanish legislation for biomedical research, the protection of personal data, and bioethics. None of the findings reported in the present study have been published elsewhere. Thos study followed the transparent reporting of a multivariable prediction model for individual prognosis or diagnosis (TRIPOD) guidelines and recent guidelines for prediction models [3,4].

Patient population

We performed a comprehensive secondary analysis of an unrestricted dataset derived from 1,241 adult (≥18 years) consecutive patients with AHRF [1] from any etiology [as defined by a PaO_2/FiO_2 ratio ≤300 mmHg on positive end-expiratory pressure (PEEP) ≥5 cmH_2O and $FiO_2 \geq 0.3$], treated with endotracheal intubation and lung-protective mechanical ventilation (MV) and conducted on 22 ICUs in

14 geographical areas of Spain, and enrolled during three periods or phases [(1 May 2017 to 30 June 2017, 1 October 2017 to 30 November 2017, and 1 February 2018 to 31 March 2018), covering the four seasons to minimize seasonal effects and variability over time]. Thus, our study only applied to adult patients receiving invasive MV with AHRF, although patients could have been on non-invasive respiratory support before intubation. In this study, the definition of AHRF did not require the presence of pulmonary or parenchymal abnormalities on chest imaging at the time of inclusion into the study, although performing chest imaging was mandatory by protocol for assessing pulmonary abnormalities at the day of study inclusion.

Based on previous work by our group [5], we focused our analysis on variables collected within the first 48 hours of AHRF diagnosis to assess the early probability of prolonged MV (defined as longer than 7 days from the time of AHRF diagnosis), independent of the underlying disease or cause of death (**Figure S1**). The unit of observation was to have collected data at the time of AHRF diagnosis (T0), at 24 hours (T24), and at 48 hours (T48). T0 was defined as the day and time at which AHRF criteria were satisfied, irrespective of date of ICU admission or initiation of MV. Patients were excluded if they were extubated or died within the first two days of AHRF diagnosis. Thus, we excluded patients that were extubated, discharged, or died before 48 hours, for which a clinician may be unlikely to use the prediction model in practice. No patients were excluded (if they met all the inclusion criteria), regardless of age, sex, underlying disease, estimated life expectancy, or duration of invasive MV. All mechanically ventilated patients had arterial blood gases at study inclusion. We did not use SpO_2 as a surrogate for PaO_2 for enrolling patients.

Study design

From a total of 1,241 patients (**Tables 1, S1**), 1,015 patients were ventilated for more than 24 hours (**Table S2**). However, we analyzed a total of 996 patients (**Tables 1, S3**), representing more than 80% of the entire cohort, after excluding 245 patients from the entire cohort of 1,241 patients, because they were ventilated <2 days and most clinical data were not collected at T48 after AHRF diagnosis (**Table S4**).

This study was conducted in several steps. *In the first step*, we analyzed baseline data from the entire cohort of 1,241 patients, and baseline data and at T48 from 996 patients with complete data at T48. Based on previous studies by our group [5], we selected risk features or clinically relevant variables at 48 hours of routine ICU management by forward and backward stepwise regression [6]. The forward

selection approach starts with no variables and adds each new variable incrementally, testing for statistical significance, while the backward elimination method begins with a full model and then removes the least statistically significant variables one at a time. We decided not to split data by hospital because the largest hospital had on average less than 25 patients in each 2-month period of the study (**Table S5**).

In the second step, we initially categorized patients into four categories, depending on whether they survived or died in the ICU and whether they were mechanically ventilated for 2 to 7 days or longer than 7 days. However, since no patients discharged alive from the ICU required readmission into the ICU, we finally grouped all survivors, independent of the duration of MV, and analyzed a total of three groups: (i) ICU survivors, (ii) non-survivors of ICU on MV for 2-7 days, (iii ICU non-survivors on MV>7 days. We analyzed baseline characteristics of ICU survivors and non-survivors of the entire cohort with AHRF. Also, we analyzed baseline (T0) and data at 48 hours in the 996 patients with complete data at T48.

Prediction models often perform poorly when assessed in validation studies [1,7,8], when studies include patients from different hospitals, different regions, different seasons, and different healthcare systems, as in our patient population.

In the third step, and with the selected, unrelated variables, we will apply different machine learning (ML) approaches with 4x10 cross-validation [Multilayer Perceptron (MLP), Random Forest (RF), Support Vector Machine (SVM)] and the multinomial regression technique (MNR) for predicting the performance of each outcome category. Cross-validation is a technique used to check how well an ML model performs on unseen data while preventing overfitting. It works by splitting the dataset into several parts, training the model on some parts and testing it on the remaining part, repeating this resampling process multiple times by choosing different parts of the dataset, and averaging the results from each validation step to achieve the final performance. The cross-validation machinery will do its best to minimize the prediction error. In our study, we repeated this approach 10 times.

A major goal of this study was predicting whether a patient belonged to one of those three groups. We selected MV for longer than 7 days as prolonged ventilation after AHRF diagnosis because the median of MV duration of 996 patients from AHRF diagnosis was 8 days (interquartile range of 4-26 days). We acknowledge that there is competitive mortality in patients on MV. Therefore, we cannot ignore ICU mortality in the prediction model. As a result, we used a regression multinomial model, with

data collected at T48. Our group has identified that after several hours of routine ICU management and therapy, the values of most baseline features and the progression of the disease process changed markedly in patients with AHRF [9]. Our primary goal was to compare the performance of several machine learning (ML) methods and conventional statistics in predicting whether a patient with AHRF could survive the ICU experience, died within a short duration of MV, or died 7 days after AHRF diagnosis.

Attending physicians recorded the cause of AHRF (or reason for MV) and comorbidities (arterial hypertension, neoplastic diseases, liver disease, cardiac disease, renal disease, immunosuppression, diabetes, neurologic disease, obesity, chronic obstructive pulmonary disease, neuromuscular disease, pregnancy, cerebrovascular disease, mental disorders, drug addiction, thyroid disease, organ transplantation, brain injury, coagulation diseases, etc.). Neoplastic diseases included cancer in solid organs and hematological malignancies. Being immunosuppressed or immunocompromised was a result of certain diseases or conditions or because of medication or treatment for a disease or condition, including, but not limited to, cancer or organ transplantation. For the purpose of our prediction model, we excluded comorbidities with a prevalence <5% (**Table S6**).

General care

Although treatment was not strictly protocolized, attending clinicians followed current guidelines for general critical care management, which included the following: (i) in case of sepsis, physicians were urged to ensure early identification of causative microorganism, intravenous administration of antibiotics as soon as bacterial sepsis was suspected or recognized, and to optimize antibiotic selection and timely administration on the bases of antibiogram; (ii) fluid resuscitation and vasopressor use were individualized with the goal of maintaining a systolic blood pressure ≥90 mmHg or a mean arterial pressure ≥65 mmHg; (iii) to maintain hemoglobin between 7 and 10 g/dL. For ventilatory management, clinicians followed the current recommendations for lung-protective ventilation, with a tidal volume (VT) of 4-8 mL/kg predicted body weight (PBW), a Pplat <30 cmH_2O, a ventilatory rate (RR) to maintain a $PaCO_2$ between 35 and 50 mmHg (permissive hypercapnia was allowed to target VT), and PEEP and FiO_2 combinations according to the PEEP-FiO_2 table of the ARDSnet protocol [10], ensuring that among the PEEP and FiO_2 combinations, clinicians should use the PEEP levels that allowed the reduction of FiO_2 to the lowest levels for maintaining a PaO_2 within a target range of 60 to 100 mmHg or to a SpO_2 within a target range of 90 to 98%.

The choice of drugs for sedation and analgesia, early neuromuscular blockade, prone positioning, hemodynamic management, and the decision to perform a tracheostomy were left to the discretion of the attending physician. PBW was calculated using the formulas of the ARDSnet protocol [10]: 50 + 0.91 x [height (cm) – 152] for men, and 45.5 + 0.91 x [height (cm) – 152] for women. Although prone positioning was used in some patients, we do not have data on the timing of prone positioning or whether prone ventilation was applied as a rescue therapy, as a routine practice, or following any specific protocol.

Weaning of MV could be started when the attending physician considered it clinically appropriate. Patients were assessed daily for readiness for a spontaneous breathing trial (SBT) based on the ARDSnet protocol [10]. In general, prerequisites for the SBT included a partial reversal of the underlying cause of AHRF, a PaO_2/FiO_2>200 mmHg with PEEP<10 cmH_2O and $FiO_2 \leq 0.4$, no vasopressors, continuous sedation minimized, and the ability to cough during tracheal aspirations. Spontaneous ventilation was tested with a T-piece or with pressure support at 8 cmH_2O. The duration of the SBT was at least 30 min and no longer than 120 min. If the patient passed the SBT, a decision for extubation was taken, unless there was a specific reason not to extubate. Weaning and the decision to extubate were left to the discretion of the responsible physician. Since the rate of reintubation after extubation for all indications is estimated at about 20% [11], for patients at high risk for reintubation [>65 years of age, hypercapnic ($PaCO_2$ >45 mmHg after extubation), or ineffective cough and excessive secretions, with ≥1 weaning failure, with more than one comorbid condition, with upper airway obstruction, or with an APACHE II score >12 on the day of extubation], non-invasive ventilatory support for 24 to 48 hours was indicated until stable or requiring reintubation [12].

Data collection and follow-up

Data were collected in each participating ICU using standardized case report forms (CRFs) and transmitted to the coordination center (Hospital Universitario Dr. Negrín, Las Palmas de Gran Canaria, Spain) when the patient was discharged from the hospital. It is important to emphasize that for this high-quality dataset, the data manager, the coordinator and principal investigator, and all local investigators from participating ICUs attended a formal session in Madrid, Spain, for reviewing and discussing the study protocol and for data collection on CRFs. All documents required for the study, including the study protocol, management guidelines, copies of CRFs, and model of an informed consent form (in case it

was needed), were available for each attending clinician at each participating ICU to ensure compliance with the daily patient screening and inclusion into the study.

Before exporting the data into a computerized database, a trained data collector from the coordinating center checked the completeness and the quality of information. Logical checks were performed for missing data and for finding inconsistences, especially regarding clinical diagnosis, comorbidities, dates, and severity scores. No information on medication or special procedures was collected. If necessary, the data collector contacted the local investigator(s) to validate the data or reformat the data for entry into the database.

We used variables including demographics, comorbidities, cause of AHRF (or reason for MV), acute physiology and chronic health evaluation II (APACHE II) score [13] during the first 24h of AHRF diagnosis, data from ventilator settings and lung mechanics [tidal volume (VT), respiratory rate (RR), positive end-expiratory pressure (PEEP), plateau pressure (Pplat)], and gas exchange [(PaO_2, $PaCO_2$, FiO_2, PaO_2/FiO_2 ratio, pH)] at T0, T24, and T48, although we focused on variables collected at 48 hours of MV after the diagnosis of AHRF to estimate the probability that a patient belonged to one of the three defined groups (ICU survivor, ICU death on MV≤7 days, and ICU death on MV>7 days). We recorded the sequential organ failure assessment (SOFA) score [14] and occurrence of extrapulmonary organ system failures (OFs) included in the SOFA scale at diagnosis of AHRF, at 24h and 48h later. Sepsis was defined by Sepsis-3 criteria [15]. Clinical suspicion of infection was defined as the co-occurrence of antibiotic administration and/or microbiological culture. We recorded the date and status (alive or dead) of patients in the ICU and hospital discharge. We recorded the occurrence of extrapulmonary OF (cardiovascular system, liver, kidney, coagulation, and central nervous system) included in the SOFA scale at diagnosis of AHRF, at T24, and at T48. Since the term “organ dysfunction” may emerge from reasons other than sepsis, extrapulmonary OF was defined as an acute change in the organ-specific SOFA score ≥2 [14,16]. Baseline SOFA was assumed to be zero in patients without preexisting organ dysfunction. We recorded the duration of MV and the length of ICU and hospital stay. Also, we recorded the date and status (alive or dead) of patients at ICU and hospital discharge and the causes of death. Since overall mortality is a composite endpoint [9], we examined death from multiple causes: pulmonary (refractory hypoxemia, tension pneumothorax, accidental ventilator disconnection), non-pulmonary (multiple system organ dysfunction, shock, terminal cancer, ventricular arrhythmias, brain death), limitation of therapeutic efforts for end-of-life, and others.

Statistical analysis plan

We defined and specified rules and expectations in advance, before the final statistical and machine learning (ML) analysis were conducted, realizing that overly detailed analyses could produce overoptimistic results due to a combination of reduced statistical power to detect real differences, due to an increase in the variance around the mean estimates, and/or an increased statistical likelihood of a false finding when many variables are examined. Since variable selection is of vital importance in building a prediction model, our aim for feature selection was to incorporate clinically relevant variables while avoiding noise/redundant variables.

First, for the purpose of our prediction model, we excluded comorbidities with a prevalence <5% (**Table S6**). *Second*, we focused our analysis on clinically relevant variables collected within the first 48 hours of diagnosis of AHRF to estimate the early probability of duration of MV>7 days, independent of the underlying disease or cause of death. Although in each patient, we recorded 246 variables during their ICU stay, we analyzed the following variables as potential early predictors of ICU outcome: age at ICU admission, sex, comorbidities, SOFA score, number of extrapulmonary OFs, PaO_2, FiO_2, PaO_2/FiO_2 ratio, $PaCO_2$, pH, FiO_2, VT, RR, PEEP, Pplat, driving pressure (calculated as the difference of Pplat minus PEEP), and minute ventilation (as an indirect measurement of dead space, and calculated as VT x RR in liters/min) all of them at T48 after AHRF diagnosis. We did not include respiratory compliance in the model because it shares collinearity with three independent variables needed for its calculation (VT, Pplat, and PEEP) and it suffers from redundancy in the descriptive model. In addition, the respiratory compliance did not contribute to the predictive validity for mortality by a panel of experts in many cases of AHRF [17]. Although we have the APACHE II score in most patients at T0 and T24, we did not include it in the model because it is a cumbersome score designed for the first 24 hours of ICU admission, it is made of 12 physiological variables and two disease-related variables, it is not routinely calculated at the bedside in most ICUs worldwide or during trial enrollment decisions, it requires numerous data elements, and it relies on laboratory data that are not uniformly collected. In addition, at least half of the variables needed to calculate the APACHE II score are included in the list of selected variables, such as age, PaO_2, FiO_2, RR, pH, renal function, neurological function, and comorbidities.

Third, although several variables shared collinearity with other independent variables (for example, the calculation of the PaO_2/FiO_2 ratio with FiO_2 and PaO_2 and Plat and PEEP for the calculation of driving pressure), we considered all variables at the initial steps of analysis. Whether driving pressure

relates causally to outcome remains to be established in multicenter randomized controlled trials [18]. Other features seemed to have redundancy.

Fourth, variable selection or feature subset selection is a common task in ML or data mining models. ML is a branch of artificial intelligence encompassing two major approaches: supervised and unsupervised learning [19]. The objective of supervised ML is to develop an algorithm capable of predicting a unique output when provided with a specific input. The expectations are that the resulting algorithm would deliver accurate predictions when exposed to new and never-before-seen data. Since the inclusion of all available variables in an ML model could produce noisy results, which are difficult to interpret, we achieved parsimony to identify a subset of relevant variables (subset selection) while excluding noise/redundant variables. We optimized the subset of selected variables by minimizing the Bayesian information criterion (BIC) [20]. BIC is a criterion for model selection among a finite set of models.

Fifth, we identified potential variables that could be included in the prediction models based on our redefined rules, their contribution to the area under the receiver operating characteristic (ROC) curve (AUC), and their p-values. The AUC is an estimator of prediction error and thereby of the relative quality of statistical models for a given set of data [21]. A ROC curve essentially has two components represented by the sensitivity and 1-specificity [21]. The AUC is an effective way to summarize the overall prognostic accuracy of a variable or test, and it is most useful for assessing the relevance of treatment effects. When representing and reporting the AUC, the point corresponding to no change (AUC=0.5) is represented by a diagonal line (45-degree line or no discriminatory ability for the outcome of interest).

Sixth, we evaluated the final model with the minimum number of variables at each time scenario using multinomial regression (MNR) and three supervised ML techniques: Random Forest (RF), Support Vector Machine (SVM), and Multilayer Perceptron (MLP) [22-26]. RF is a supervised ML algorithm that combines the output of multiple decision trees to reach a single result. The RF algorithm is made up of a collection of decision trees, and each tree in the ensemble comprises a data sample drawn from a training set with a replacement [24]. While decision trees consider all the possible feature splits, RF only selects a subset of those features, resulting in precise predictions. The SVM is one of the prevailing algorithms because the data in biomedical research are often limited. One of the main strengths of the SVM is the ability to efficiently construct complex decision boundaries from limited samples [22,25]. The

deep learning MLP is a feed-forward neural network with a basic architecture comprising fully connected layers [25,26]. The input layer has the same number of inputs as the total predictor variables. The middle layer looks for characteristics associated with the data. The output layer has the same number of outputs as the categories to predict.

Calculations were performed using the R Core Team software 20243 (R version 4.5.0) (https://www.r-project.org) (R Foundation for Statistical Computing, Vienna, Austria). We compared the predictive performance of the three ML methods using the following parameters: accuracy, sensitivity, specificity, true positive, false positive, false negative, and true negative [27].

Data analysis

We calculated the mean, standard deviation (SD), median, and interquartile range (IQR) of the quantitative variables. We used the Kolmogorov–Smirnov test to examine the normal distribution of data. We calculated the frequency and percentage of qualitative variables. We reported data as percentages or mean ± SD, unless otherwise specified. We reported the odds ratio and 95% confidence intervals (CIs). We assessed differences in the values of clinically relevant features in the three scenarios. We analyzed differences between the distributions of categorical variables with Fisher's exact test. We identified potential variables that could be included in the prediction model based on our predefined rules, the AUC, and their p-values. For all comparisons, a two-sided significance level of p-value <0.005 was considered a real effect size, as recommended [28].

SUPPLEMENTARY RESULTS

From the total cohort of 1,241 patients with AHRF, 438 patients (35.3%) died in the ICU: 111 (8.9%) in less than 48 h of MV from AHRF diagnosis, 184 (14.8%) with 2-7 days on MV, and 143 (11.5) patients on MV >7 days (**Figure 1**). A total of 111 patients died in the ICU at <48 hours of AHRF diagnosis: the major causes or reasons for MV were sepsis/pancreatitis and post-cardiac arrest, and the major cause of death was multiple system organ failure (**Table S4**).

A total of 996 patients, representing more than 80% of the entire cohort, were ventilated for ≥48 hours, and the number of ICU deaths in each phase of the study was similar [116/337 (34.4%), 96/313 (30.7%), 115/346 (33.2%) (p=0.586)]. Overall, most patients (n=503) were ventilated for >7 days, although the number of ICU deaths (n=143) was relatively low (28.4%). When referring to 996 patients with data at T48, each phase of the study had more than 300 patients (n=337, 313, 346, respectively), and their ICU mortality was similar (p=0.586) (**Table S5**).

The median of days since the last day of MV to discharge from the ICU in the entire population of 1,241 and the cohort of 996 was similar (4.2±7.9 vs. 4.6±8.4 days) (p=0.247).

The most common comorbidities in 996 patients were arterial hypertension (50.5%), diabetes (27.7%), and obesity (22.7%) (**Table S6**). As planned, in the prediction model, we only included those comorbidities with a prevalence >5% (n=at least 50 patients). When considering the entire cohort, independent of the duration of MV, a total of 803 patients (64.7%) survived at ICU discharge. Only 145 patients died in the ICU requiring MV>7 days (**Table S7**), most of them had pneumonia (20.0%) and were severe post-surgical patients (18.6%) and required a higher mean PEEP to maintain a PaO_2/FiO_2 >100-150 mmHg and a higher minute ventilation to maintain a $PaCO_2$ <50 mmHg.

From the cohort of 996 patients with AHRF and complete data at 48 hours on MV, baseline values and T48 values of gas exchange, lung mechanics, and severity scores varied significantly within 48 hours of management. Most of them (n=669) were discharged alive (67.2%) from the ICU, and only 143 (14.4%) died in the ICU after being on MV >7 days. (**Tables S8, S9**). Of those 143 patients, 29 patients (20.3%) had pneumonia, 26 patients (18.2%) were severe post-surgical, and 17 patients (11.9%) had severe stroke/coma.

SUPPLEMENTARY DISCUSSION

In AHRF patients with such heterogeneous clinical etiologies or reasons for MV, it is reasonable to expect that with a simple clinical syndrome based only on gas exchange, the effect of several different therapies on outcomes may differ with variable characteristics [29]. Individualized treatment in AHRF does not influence another individual's potential outcomes [30]. We believe that a definition of AHRF based only on gas exchange is useless, especially when the presence of pulmonary or parenchymal abnormalities on chest imaging at the time of diagnosis is not required. Almost all critically ill patients could meet the gas-exchange criteria for AHRF on MV. Although our data adequately represents the range of clinical contexts encountered at future hospitals, AHRF arises as a response to various underlying and unrelated conditions (sepsis, multiple traumas, aspiration pneumonia, head trauma, post-cardiac arrest, etc.). It is a complex and challenging syndrome to define precisely due to its multifactorial nature with an ample range of outcomes [9,31].

In addition to a definition based on gas exchange (PaO_2, FiO_2, PEEP), the challenges for a needed AHRF definition include data availability and quality, lack of standardization, adaptability to real clinical settings, trust and acceptance, ethical concerns [32], regulatory hurdles [33] (where readmission shortly after an initial ICU or hospital stay could be considered to indicate suboptimal care, separate etiologies or reasons for MV (otherwise we will not know on which etiology our therapeutic efforts to reduce mortality are more beneficial), precise definitions of parenchymal abnormalities, and a lack of gold standard diagnosis and management for AHRF.

For the duration of MV, timely and safe extubation is vitally important to avoid increased morbidity, costs, ICY stays, and deaths. Although spontaneous breathing trials (SBTs) are regarded internationally as the standard of care to assess liberation from MV, little is known about predictors of extubation success [34]. Most ventilated critically ill patients undergo at least a trial of extubation. We need to innovate to combine clinicians' assessment of extubation readiness with predictive analytics. There is growing evidence changing current clinical practice and supporting strategies to liberate critically ill patients by integrating individual patient data to aid clinicians with extubation decision making and improve outcomes [35]. However, evidence to support extubation decision making is lacking. Since weaning practices vary internationally, we need hybrid consent models for the better prediction of liberation from MV and extubation outcomes.

We acknowledge that most models lack external and prospective validation. Real-world clinical settings often exhibit variations in patient demographics, treatment protocols, and disease prevalence. Therefore, a rigorous validation and adaptation process is necessary to ensure the robustness and reliability of prediction models.

TABLE S1. Cumulative number of patients in the observational pooled cohort of 1,241 patients with acute hypoxemic respiratory failure (AHRF) in relation to number of days on mechanical ventilation from the diagnosis of AHRF and status (survivor/dead) at ICU discharge. Highlighted rows in yellow represent selected ICU outcomes in this study (7-day, 28-day, 60-day ICU discharge). The annotation “>60” represents ICU discharge.

Days on MV	Cumulative No. of patients	Cumulative ICU survivors	Cumulative ICU deaths	Proportion of survivors	Proportion of deaths (%)	Difference in proportions 95% CI
1	226	125	101	55.3	44.7	10.6 % (6.7 to 14.5)
2	372	215	157	57.8	42.2	13.6 % (9.7 to 17.5)
3	475	272	203	57.3	42.7	14.6% (10.7 to 18.5)
4	551	320	231	58.1	41.9	16.2% (12.3 to 20.0)
5	610	357	253	58.5	41.5	16.2% (12.3 to 20.0)
6	659	393	266	59.6	40.4	18.2% (14.3 to 22.0)
7	733	440	293	60.0	40.0	20.2% (16.1 to 23.8)
8	784	475	309	60.6	39.4	21.2% (17.3 to 25.0)
9	820	501	319	61.1	38.9	22.2% (18.3 to 26.0)
10	851	526	325	61.8	38.2	23.6% (19.7 to 27.4)
11	886	551	335	62.2	37.8	24.4% (20.5 to 28.2)
12	914	570	344	62.4	37.6	24.8% (20.9 to 28.6)
13	929	583	346	62.8	37.2	25.6% (21.7 to 29.3)
14	961	603	358	62.8	37.2	25.6% (21.7 to 29.3)
15	978	616	362	63.0	37.0	26.0% (22.1 to 29.7)
16	994	629	365	63.3	36.7	26.6% (22.8 to 30.3)
17	1019	652	367	64.0	36.0	28.0% (24.2 to 31.7)
18	1034	663	371	64.1	35.9	28.2% (24.4 to 31.9)
19	1049	673	376	64.2	35.8	28.4% (24.6 to 32.1)
20	1060	682	378	64.3	35.7	28.6% (24.8 to 32.3)
21	1085	703	382	64.8	35.2	29.6% (25.8 to 33.3)
22	1094	709	385	64.8	35.2	29.6% (25.8 to 33.3)
23	1107	719	388	65.0	35.1	29.2% (26.1 to 33.6)
24	1120	730	390	65.2	34.8	30.4% (26.6 to 34.1)
25	1128	734	394	65.1	34.9	30.2% (26.4 to 33.9)
26	1139	741	398	65.1	34.9	30.2% (26.4 to 33.9)
27	1149	748	401	65.1	34.9	30.2% (26.4 to 33.9)
28	1161	755	406	65.0	35.0	30.0% (26.2 to 33.7)
29	1167	760	407	65.1	34.9	30.2% (26.4 to 33.9)
30	1172	764	408	65.2	34.8	30.4% (26.6 to 34.1)
31	1181	768	413	65.0	35.0	30.0% (26.2 to 33.7)
32	1184	771	413	65.1	34.9	30.2% (26.4 to 33.9)
33	1185	771	414	65.1	34.9	30.2% (26.4 to 33.9)
34	1189	773	416	65.0	35.0	30.0% (26.2 to 33.7)
35	1193	775	418	65.0	35.0	30.0% (26.2 to 33.7)
36	1196	777	419	65.0	35.0	30.0% (26.2 to 33.7)
37	1199	778	421	64.9	35.1	29.8% (26.o to 33.5)
38	1202	779	423	64.8	35.2	29.6% (25.8 to 33.3)
39	1203	780	423	64.8	35.2	29.6% (25.8 to 33.3)
40	1204	781	423	64.9	35.1	29.8% (26.0 to 33.5)
41	1204	781	423	64.9	35.1	29.8% (26.0 to 33.5)
42	1208	783	425	64.8	35.2	29.6% (25.8 to 33.3)
43	1208	783	425	64.8	35.2	29.6% (25.8 to 33.3)

44	1209	783	426	64.8	35.2	29.6% (25.8 to 33.3)
45	1213	785	428	64.7	35.3	29.4% (25.6 to 33.1)
46	1214	786	428	64.7	35.3	29.4% (25.6 to 33.1)
47	1215	787	428	64.8	35.2	29.6% (25.8 to 33.3)
48	1218	789	429	64.8	35.2	29.6% (25.8 to 33.3)
49	1219	790	429	64.8	35.2	29.6% (25.8 to 33.3)
50	1220	791	429	64.8	35.2	29.6% (25.8 to 33.3)
51	1223	793	430	64.8	35.2	29.6% (25.8 to 33.3)
52	1224	793	431	64.8	35.2	29.6% (25.8 to 33.3)
53	1224	793	431	64.8	35.2	29.6% (25.8 to 33.3)
54	1226	794	432	64.8	35.2	29.6% (25.8 to 33.3)
55	1227	794	433	64.7	35.3	29.4% (25.6 to 33.1)
56	1229	795	434	64.7	35.3	29.4% (25.6 to 33.1)
57	1230	796	434	64.7	35.3	29.4% (25.6 to 33.1)
58	1231	797	434	64.7	35.3	29.4% (25.6 to 33.1)
59	1231	797	434	64.7	35.3	29.4% (25.6 to 33.1)
60	1231	797	434	64.7	35.3	29.4% (25.6 to 33.1)
>60	1241	803	438	64.7	35.3	29.4% (25.6 to 33.1)

AHRF: acute hypoxemic respiratory distress failure; CI: confidence interval; ICU: intensive care unit; MV: mechanical ventilation; No.: number.

TABLE S2. Cumulative number of patients in the observational pooled cohort of 1,015 patients with acute hypoxemic respiratory failure (AHRF) mechanically ventilated for more than 24 hours, in relation to the number of days on mechanical ventilation (MV) from diagnosis of AHRF and status (survivor/dead) at ICU discharge. Highlighted rows in yellow represent selected ICU outcomes in this study (7-day, 28-day, 60-day ICU discharge). The annotation “>60” represents ICU discharge.

Days on MV	Cumulative No. of patients	Cumulative ICU survivors	Cumulative ICU deaths	Proportion of survivors	Proportion of deaths (%)	Difference in proportions 95% CI
2	146	90	56	61.6	38.4	23.2 (18.9 to 27.4)
3	249	147	102	59.0	41.0	18.0 (13.7 to 22.2)
4	325	195	130	60.0	40.0	20.0 (15.7 to 24.2)
5	384	232	152	60.4	39.6	28.8 (16.5 to 25.0)
6	433	268	165	61.9	38.1	23.8 (19.5 to 28.0)
7	507	315	192	62.1	37.9	24.2 (19.9 to 28.3)
8	558	350	208	62.7	37.3	25.4 (21.1 to 29.5)
9	594	376	218	63.3	36.7	26.2 (22.3 to 30.7)
10	625	401	224	64.2	35.8	28.4 (24.2 to 32.5)
11	660	426	234	64.6	35.5	29.1 (24.9 to 33.2)
12	688	445	243	64.7	35.3	29.4 (25.2 to 33.5)
13	703	458	245	65.2	34.9	30.3 (26.1 to 34.4)
14	735	478	257	65.0	35.0	30.0 (25.8 to 34.1)
15	752	491	261	65.3	34.7	30.6 (26.4 to 34.7)
16	768	504	264	65.6	34.4	31.2 (27.0 to 35.2)
17	793	527	266	66.5	33.5	33.0 (28.8 to 37.0)
18	808	538	270	66.6	33.4	32.2 (28.0 to 36.2)
19	823	548	275	66.6	33.4	32.2 (28.0 to 36.2)
20	834	557	277	66.8	33.2	33.6 (29.4 to 37.6)
21	859	578	281	67.3	32.7	34.6 (30.4 to 38.6)
22	868	584	284	67.3	32.7	34.6 (30.4 to 38.6)
23	881	594	287	67.4	32.6	34.8 (30.6 to 38.8)
24	894	605	289	67.7	32.3	35.4 (31.2 to 39.4)
25	902	609	293	67.5	32.5	35.0 (30.8 to 39.0)
26	913	616	297	67.5	32.5	35.0 (30.8 to 39.0)
27	923	623	300	67.5	32.5	35.0 (30.8 to 39.0)
28	935	630	305	67.4	32.6	34.8 (30.6 to 38.8)
29	941	635	306	67.5	32.5	35.0 (30.8 to 39.0)
30	946	639	307	67.6	32.5	35.1 (30.9 to 39.1)
31	955	643	312	67.3	32.7	34.6 (30.4 to 38.6)
32	958	646	312	67.4	32.6	34.8 (30.6 to 38.3)
33	959	646	313	67.4	32.6	34.8 (30.6 to 38.3)
34	963	648	315	67.3	32.7	34.6 (30.4 to 38.6)
35	967	650	317	67.2	32.8	30.4 (30.2 to 38.4)
36	970	652	318	67.2	32.8	30.4 (30.2 to 38.4)
37	973	653	320	67.1	32.9	34.2 (30.0 to 38.2)
38	976	654	322	67.0	33.0	34.0 (29.8 to 38.0)
39	977	655	322	67.0	33.0	34.0 (29.8 to 38.0)
40	978	656	322	67.1	32.9	34.2 (30.0 to 38.2)
41	978	656	322	67.1	32.9	34.2 (30.0 to 38.2)
42	982	658	324	67.0	33.0	34.0 (29.8 to 38.0)
43	982	658	324	67.0	33.0	34.0 (29.8 to 38.0)
44	983	658	325	66.9	33.1	33.8 (29.6 to 37.8)

45	987	660	327	66.9	33.1	33.8 (29.6 to 37.8)
46	988	661	327	66.9	33.1	33.8 (29.6 to 37.8)
47	989	662	327	66.9	33.1	33.8 (29.6 to 37.8)
48	992	664	328	66.9	33.1	33.8 (29.6 to 37.8)
49	993	665	328	67.0	33.0	34.0 (29.8 to 38.0)
50	994	666	328	67.0	33.0	34.0 (29.8 to 38.0)
51	997	668	329	67.0	33.0	34.0 (29.8 to 38.0)
52	998	668	330	66.9	33.1	33.8 (29.6 to 37.8)
53	998	668	330	66.9	33.1	33.8 (29.6 to 37.8)
54	1000	669	331	66.9	33.1	33.8 (29.6 to 37.8)
55	1001	669	332	66.8	33.2	33.6 (29.4 to 37.6)
56	1003	670	333	66.8	33.2	33.6 (29.4 to 37.6)
57	1004	671	333	66.8	33.2	33.6 (29.4 to 37.6)
58	1005	672	333	66.9	33.1	33.8 (29.6 to 37.8)
59	1005	672	333	66.9	33.1	33.8 (29.6 to 37.8)
60	1005	672	333	66.9	33.1	33.8 (29.6 to 37.8)
>60	1015	678	337	66.8	33.2	33.6 (29.4 to 37.6)

AHRF: acute hypoxemic respiratory distress failure; CI: confidence interval; ICU: intensive care unit; MV: mechanical ventilation; No.: number.

TABLE S3. Cumulative number of patients in the observational pooled cohort of 996 patients with acute hypoxemic respiratory failure (AHRF), mechanically ventilated for at least 48 hours, with complete data at 48 hours after AHRF diagnosis, in relation to number of days on ventilatory support and status (survivor/dead) at ICU discharge from the time of AHRF diagnosis. Highlighted rows in yellow represent selected ICU outcomes in this study (7-day, 28-day, 60-day, >60). The annotation ">60" represents ICU discharge.

Days on MV	Cumulative No. of patients	Cumulative ICU survivors	Cumulative ICU deaths	Proportion of survivors	Proportion of deaths (%)	Difference in proportions 95% CI
2	133	84	49	63.2	36.8	26.4 ((22.1 to 30.6)
3	236	141	95	59,8	40.3	19.5 (15.1 to 23.7)
4	312	189	123	60.6	39.4	21.2 (16.9 to 25.4)
5	371	226	145	60.9	39.1	21.8 ((17.5 to 26.0)
6	419	262	157	62.5	37.5	25.0 (20.7 to 29.2)
7	493	309	184	62.7	37.3	25.4 (21.1 to 29.6)
8	544	344	200	63.2	36.8	26.4 (22.1 to 30.6)
9	579	369	210	63.7	36.3	27.4 (23.1 to 31.5)
10	610	394	216	64.6	35.4	29.2 (24.9 to 33.3)
11	645	419	226	65.0	35.0	30.0 (25.7 to 34.1)
12	673	438	235	65.1	34.9	30.2 (25.9 to 34.3)
13	688	451	237	65.6	34.5	31.1 (26.8 to 35.2)
14	720	471	249	65.4	34.6	30.8 (26.5 to 34.9)
15	737	484	253	65.7	34.3	31.4 (27.3 to 35.5)
16	753	497	256	66.0	34.0	32.0 (27.8 to 36.1)
17	778	520	258	66.8	33.2	33.6 (29.4 to 37.6)
18	793	531	262	67.0	33.0	34.0 (29.8 to 38.0)
19	807	541	266	67.0	33.0	34.0 (29.8 to 38.0)
20	818	550	268	67.2	32.8	34.4 (30.2 to 38.4)
21	842	570	272	67.7	32.3	35.4 (31.2 to 39.4)
22	851	576	275	67.7	32.3	35.4 (31.2 to 39.4)
23	864	586	278	67.8	32.2	35.6 (31.4 to 39.6)
24	877	597	280	68.1	31.9	36.2 (32.0 to 40.2)
25	885	601	284	67.9	32.1	35.8 (31.6 to 39.8)
26	896	608	288	67.9	32.1	35.8 (31.6 to 39.8)
27	906	615	291	67.9	32.1	35.8 (31.6 to 39.8)
28	918	622	296	67.8	32.2	35.6 (31.4 to 39.6)
29	924	627	297	67.9	32.1	35.8 (31.6 to 39.8)
30	929	631	298	67.9	32.1	35.8 (31.6 to 39.8)
31	937	635	302	67.8	32.2	35.6 (31.4 to 39.6)
32	940	638	302	67.9	32.1	35.8 (31.6 to 39.8)
33	941	638	303	67.8	32.2	35.6 (31.4 to 39.6)
34	945	640	305	67.7	32.3	35.4 (31.2 to 39.4)
35	949	642	307	67.7	32.4	35.3 (31.1 to 39.3)
36	952	644	308	67.7	32.4	35.3 (31.1 to 39.3)
37	955	645	310	67.5	32.5	35.0 (30.8 to 39.0)
38	958	646	312	67.4	32.6	34.8 (30.6 to 38.8)
39	959	647	312	67.5	32.5	35.0 (30.8 to 39.0)
40	960	648	312	67.5	32.5	35.0 (30.8 to 39.0)
41	960	648	312	67.5	32.5	35.0 (30.8 to 39.0)

42	964	650	314	67.4	32.6	34.8 (30.6 to 38.8)
43	964	650	314	67.4	32.6	34.8 (30.6 to 38.8)
44	965	650	315	67.4	32.6	34.8 (30.6 to 38.8)
45	969	652	317	67.3	32.7	34.6 (30.4 to 38.6)
46	970	653	317	67.3	32.7	34.6 (30.4 to 38.6)
47	971	654	317	67.4	32.7	34.7 (30.5 to 38.7)
48	974	656	318	67.4	32.7	34.7 (30.5 to 38.7)
49	975	657	318	67.4	32.6	34.8 (30.6 to 38.3)
50	976	658	318	67.4	32.6	34.8 (30.6 to 38.8)
51	979	660	319	67.4	32.6	34.8 (30.6 to 38.3)
52	980	660	320	67.4	32.7	34.7 (30.5 to 38.7)
53	980	660	320	67.4	32.7	34.7 (30.5 to 38.7)
54	982	661	321	67.3	32.7	34.6 (30.4 to 38.6)
55	983	661	322	67.2	32.8	34.4 (30.2 to 38.4)
56	985	662	323	67.2	32.8	34.4 (30.2 to 38.4)
57	986	663	323	67.2	32.8	34.4 (30.2 to 38.4)
58	987	664	323	67.3	32.7	34.6 (30.4 to 38.6)
59	987	664	323	67.3	32.7	34.6 (30.4 to 38.6)
60	987	664	323	67.3	32.7	34.6 (30.4 to 38.6)
>60	996	669	327	67.2	32.8	34.4 (30.2 to 38.4)

AHRF: acute hypoxemic respiratory distress failure; CI: confidence interval; ICU: intensive care unit; MV: mechanical ventilation; No.: number.

TABLE S4. Two-hundred and forty-five excluded patients with acute hypoxemic respiratory failure (AHRF) with no data at 48h. Causes of AHRF in 245 patients and causes of death in 111 patients (45.3%) who died within the first 48 hours of AHRF.

Number of patients with no data at 48 hours	N (%)	No. of ICU deaths at <48 hours of AHRF diagnosis (%) *
Etiology (reasons for invasive MV)		
Post-surgery	64 (26.1)	14 (12.6)
Cardiac arrest	29 (11.8)	20 (18.0)
Sepsis/acute pancreatitis	34 (13.9)	28 (25.2)
Cardiac failure/fluid overload	13 (5.3)	3 (2.7)
Pneumonia	20 (8.2)	13 (11.7)
Stroke or coma	29 (11.8)	14 (12.6)
Aspiration/inhalation	4 (1.6)	2 (1.8)
Trauma	16 (6.5)	10 (9.0)
Others	36 (14.7)	7 (6.3)
Total	245	111 (45.3)
Causes of death, N (%) *		
Multiple system organ failure	-	41 (36.9)
Irreversible shock	-	24 (21.6)
Brain death	-	19 (17.1)
Limitations of therapeutic efforts	-	16 (14.4)
Cardiac arrhythmia	-	6 (5.4)
Refractory hypoxemia	-	5 (4.5)

***Abbreviations**: (*) rate of death was calculated based on the total number of deaths (n=111); AHRF: acute hypoxemic respiratory failure; ICU: intensive care unit; MV: mechanical ventilation; N: number of patients.*

TABLE S5. Baseline characteristics and outcome data of 996 patients with acute hypoxemic respiratory failure (AHRF) on mechanical ventilation distributed in the three phases of the study.

Variables	Phase I N=337	Phase II N=313	Phase III N=346	p-value
Age, years, median (IQR)	65 (52-73)	65 (54-74)	64 (53-72)	0.604
Age, years, mean±SD	61.8±15.2	62.8±14.2	61.8±14.3	0.604
Sex, *n (%)*				0.905
Male	227 (67.4)	215 (68.7)	238 (68.8)	
Female	110 (32.6)	98 (31.3)	108 (31.2)	
Etiology (reasons for invasive MV), *n* (%)				
Post-surgery	56 (16.6)	47 (15.0)	41 (11.9)	0.197
Stroke or coma	53 (15.7)	57 (18.2)	52 (15.0)	0.514
Pneumonia	36 (10.7)	44 (14.1)	69 (19.9)	0.003
Sepsis/Acute Pancreatitis	46 (13.7)	33 (10.5)	39 (11.3)	0.434
Trauma	53 (15.7)	51 (16.3)	31 (9.0)	0.008
Cardiac arrest	32 (9.5)	22 (7.0)	34 (9.8)	0.393
Cardiac failure/fluid overload	14 (4.2)	18 (5.8)	17 (4.9)	0.644
Aspiration/Inhalation	17 (5.0)	16 (5.1)	12 (3.5)	0.507
Others	28 (8.3)	25 (8.0)	48 (13.9)	0.017
Unknown etiology	2 (0.6)	0 (0)	3 (0.9)	0.009
APACHE II score, mean±SD §	21.0±7.4	20.7±7.5	20.5±7.7	0.684
SOFA score, mean±SD	9.3±3.4	8.8±3.2	8.7±3.2	0.039
FiO_2, mean±SD	0.65±0.23	0.61±0.20	0.64±0.22	0.053
PaO_2, mmHg, mean±SD	99.1±33.3	100.4±36.8	97.0±34.4	0.444
PaO_2/FiO_2, mmHg, mean±SD	168.1±64.9	176.8±62.7	165.4±66.0	0.064
$PaCO_2$, mmHg, mean±SD	45.7±11.6	45.4±11.3	47.0±13.2	0.190
pH, mean±SD	7.32±0.10	7.33±0.10	7.32±0.11	0.368
VT, mL/kg PBW, mean±SD	6.89±1.10	6.92±1.14	6.91±0.95	1.0
Respiratory rate, ventilator cycles/min, mean±SD	19.7±4.4	19.9±4.2	20.1±4.6	0.496
Minute ventilation, L/min, mean±SD	8.6±2.1	8.7±1.9	8.8±2.1	0.440
PEEP, cmH_2O, mean±SD	7.8±2.8	7.8±2.7	8.2±3.0	0.107
Plateau pressure, cmH_2O, mean±SD	22.3±5.3	22.2±5.1	22.2±5.7	0.893
Driving pressure, cmH_2O, mean±SD	14.5±4.5	14.5±4.5	14.0±5.0	0.276
No. extrapulmonary OF, mean±SD	1.82±1.07	1.65±0.95	1.64±0.99	0.042
Length of ICU stay, d, median (IQR)	12 (7-22)	15 (7-27)	12 (7-22)	0.306
Days from last day MV to ICU discharge, median (IQR)	2 (0-6)	3 (0-7)	2 (0-5)	0.533
All-cause ICU mortality, n (%: 95%CI)	116 (34.4)	96 (30.7)	115 (33.2)	0.586
All-cause hospital mortality, n (%: 95%CI)	138 (40.9)	120 (38.3)	135 (39.0)	0.775

APACHE: acute physiology and chronic health evaluation; C: confidence interval; d: days; FiO_2: fraction of inspired oxygen concentration; ICU: intensive care unit; IQR: interquartile range; MV: mechanical ventilation; OF: organ failure; PBW: predicted body weight; PEEP: positive end-expiratory pressure; SD: standard deviation; SOFA: sequential organ failure assessment scale; VT: tidal volume.

§ APACHE II was not reported at baseline in 7 patients from phase I, 17 patients from phase II, and 11 patients from phase III.

TABLE S6: Comorbidities in 996 ventilated patients with acute hypoxemic respiratory failure (AHRF). *Note: Some patients could have more than 1 comorbidity.*

Comorbidities	Total (N=996) No. (%) [95% CI]	ICU survivors (N=669) n (%) [95% CI]	ICU non-survivors (N=327) n (%) [95% CI]
Arterial hypertension	503 (50.5) [47.4 to 53.6]	312 (62.0) [57,8 to 66.3]	191 (38.0) [33.7 to 42.2]
Diabetes	276 (27.7) [24.9 to 30.5]	168 (60.9) [55.1to 66.6]	108 (39.1) [33.4 to 44.9]
Obesity	226 (22.7) [20.1 to 25.3]	154 (68.1) [62.1to 74.2]	72 (39.1) [25.8 to 37.9]
COPD	162 (16.3) [14.0 to 18.6]	102 (63.0) [55.5 to 70.4]	60 (37.0) [29.6 to 44,5]
Cardiac failure	151 (15.2) [12.9 to 17.4]	89 (58.9) [51,1 to 66.8]	62 (41.1) [33.2 to 48.9]
Neoplastic diseases	136 (13.7) [11.5 to 15.8]	74 (54.4) [46.0 to 62,8]	62 (45.6) [37.2 to 54.0]
Immunosuppressed	97 (9.7) [7.9 to 11.6]	52 (53.6) [43.7 to 63.5]	45 (46.4) [36,5 to 56.3]
Chronic renal disease	80 (8.0) [6.3 to 9.7]	43 (53.8) [42.8 to 64.7]	37 (46.3) [35.3 to 57.2]
Chronic liver failure	49 (4.9) [3.6 to 6.3]	26 (53.1) [39.1 to 67.0]	23 (46.9) [330.0 to 60.9]
Coronary artery disease	44 (4.4) [3.1 to 5.7]	30 (68.2) [54.4 to 81.9]	14 (31.8) [18.1 to 45.6]
Other chronic pulmonary diseases	44 (4.4) [3.1 to 5.7]	26 (59.1) [44.6 to 73.6]	18 (40.9) [26.4 to 55.4]
Non-invasive MV at home	38 (3.8) [2.6 to 5.0]	25 (65.8) [50.7 to 80.9]	13 (34.2) [19.1 to 49.3]
Neurological disease	30 (3.0) [2.0 to 4.1]	17 (56.7) [38.9 to 74.4]	13 (43.3) [25.6 to 61.1]
Mental disorders	30 (3.0) [2.o to 4.1]	25 (83.3) [70.0 to 96.7]	5 (16.7) [3,3 to 30.0]
Thyroid disease	28 (2.8) [1.8 to 3.8]	19 (67.9) 50.6[to 85.2]	9 (32.1) [14.8 to 49.4]
Cerebrovascular disease	21 (2.1) [1.2 to 3.0]	13 (61.9) 41.1[to 82.7]	8 (38.1) [17.3 to 58.9]
Neuromuscular disease	22 (2.2) [1.3 to 3.1]	16 (72.7) [54.1 to 91.3]	6 (27.3) [8.7 to 45.9]
Organ transplantation	9 (0.9) [0.3 to 1.5]	0 (0) [0.0 to 33.6]	9 (100.0) [66.4 to 100.0]
Others	25 (2.5) [1.5 to 3.5]	18 (72.0) [54.4 to 89.6]	7 (28.0) [10.4 to 45.6]
Patients with no comorbidities reported	182 (18.3) [15.9 to 20.7]	153 (84.1) [78.8 to 89.4]	29 (15.9) [10.6 to 21.3]

Note: Only the comorbidities with ≥5% of patients (≥**50** patients) were considered for the prediction model. *Abbreviations: CI: confidence interval; COPD: chronic obstructive pulmonary disease; ICU: intensive care unit. MV: mechanical ventilation.*

TABLE S7. Baseline characteristics (T0) of 1,241 patients with acute hypoxemic respiratory failure (AHRF) in relation to ICU outcome (survivor or dead) and duration of mechanical ventilation (≤7 days vs. >7 days).

**Variables**	**ICU survivor** (independent of MV duration)	**ICU death on MV 2-7 days**	**ICU death on MV >7 days**
N, n (%)	803 (64.7)	293 (23.6)	145 (11.7)
Age, yr.	61.0 ± 14.7	66.8 ± 12.5	65.6 ± 13.1
Sex, n (%)			
female	256 (31.9)	110 (37.5)	41 (28.3)
male	547 (68.1)	183 (62.5)	104 (71.7)
Comorbidities, n (%)			
Arterial hypertension	391 (48.7)	177 (60.4)	81 (55.9)
Diabetes	206 (25.7)	81 (27.6)	55 (37.9)
Obesity	170 (21.2)	63 (21.5)	32 (22.1)
COPD	122 (15.2)	48 (16.4)	30 (20.7)
Cardiac failure	111 (13.8)	49 (16.7)	30 (20.7)
Neoplastic diseases	91 (11.3)	52 (17.7)	33 (22.8)
Immunosuppressed	63 (7.8)	46 (15.7)	22 (15.2)
Chronic renal disease	50 (6.2)	27 (9.2)	21 (14.5)
Reasons for MV, n (%)			
Post-surgery	141 (17.6)	40 (13.7)	27 (18.6)
Cardiac arrest	47 (5.9)	58 (19.8)	12 (8.3)
Sepsis/acute pancreatitis	83 (10.3)	52 (17.7)	17 (11.7)
Cardiac failure/fluid overload	39 (5.9)	12 (4.1)	11 (7.6)
Pneumonia	45 (4.9)	38 (13.0)	29 (20.0)
Stroke or coma	112 (15.2)	52 (17.7)	17 (11.7)
Aspiration/inhalation	38 (4.7)	4 (1.4)	7 (4.8)
Trauma	123 (15.3)	19 (6.5)	9 (6.2)
Others	108 (13.4)	18 (6.1)	16 (11.0)
SOFA	8.1 ± 3.2	11.0 ± 3.4	9.8 ± 3.4
VT, *ml/kg PBW*	6.9 ± 1.0	6.7 ± 1.1	6.8 ± 1.2
FiO_2	0.62 ± 0.21	0.66 ± 0.23	0.66 ± 0.21
Respiratory rate, *cycles/min*	19.5 ± 4.2	19.8 ± 4.5	20.9 ± 5.1
PEEP, *cmH_2O*	7.8 ± 2.8	7.5 ± 2.6	8.4 ± 2.9
Plateau pressure, *cmH_2O*	21.0 ± 4.7	24.7 ± 6.1	24.9 ± 5.7
Driving pressure, *cmH_2O*	13.2 ± 3.9	17.1 ± 5.6	16.5 ± 5.1
PaO_2, *mmHg*	99.4 ± 34.7	98.4 ± 34.5	97.5 ± 34.1
PaO_2/FiO_2, *mmHg*	173.7 ± 61.4	165.4 ±68.1	163.3 ±69.6
$PaCO_2$, *mmHg*	45.9 ± 11.7	46.2 ± 14.0	47.4 ± 12.8
pH	7.33 ± 0.10	7.29 ± 0.14	7.30 ± 0.11
No. extrapulmonary OF	1.5 ± 1.0	2.3 ± 1.0	2.0 ± 1.1
Minute ventilation, L/min	8.6 ± 2.1	8.5 ± 2.1	8.8 ± 2.1

Values are reported as means ± standard deviation (SD). ***Abbreviations****: AHRF: acute hypoxemic respiratory failure, bpm: breaths per minute, d: days, ICU: intensive care unit, MV: mechanical ventilation, N/n/no: number of patients, OF: organ failure, PBW: predicted body weight, SOFA: sequential organ dysfunction assessment, T0: at the time of AHRF diagnosis.*

TABLE S8. Baseline characteristics of 996 patients with acute hypoxemic respiratory failure (AHRF) mechanically ventilated for ≥2 days in relation to ICU outcome (survivor or dead) and duration of mechanical ventilation (2-7 days vs. >7 days).

**Variables**	**ICU survivor** (independent of MV duration)	**ICU death on MV 2-7 days**	**ICU death on MV>7 days**
N, n (%)	669 (67.2)	184 (18.5)	143 (14.4)
Age, yr.	60.2 ± 14.9	66.2 ± 12.9	65.7 ± 13.2
Sex, n (%)			
female	210 (31.4)	67 (36.4)	39 (27.3)
male	459 (68.6)	117 (63.6)	104 (72.7)
Comorbidities, n (%)			
Arterial hypertension	312 (46.6)	110 (59.8)	81 (56.6)
Diabetes	168 (25.1)	53 (28.8)	55 (38.5)
Obesity	134 (20.0)	40 (21.7)	32 (22.4)
COPD	102 (15.2)	30 (16.3)	30 (21.0)
Cardiac failure	89 (13.3)	32 (17.4)	30 (21.0)
Neoplastic diseases	74 (11.1)	30 (16.3)	32 (22.4)
Immunosuppressed	52 (7.8)	23 (12.5)	22 (15.4)
Chronic renal disease	43 (6.4)	16 (8.7)	21 (14.7)
Reasons for MV, n (%)			
Post-surgery	91 (13.6)	27 (14.7)	26 (18.2)
Cardiac arrest	38 (5.7)	38 (20.7)	12 (8.4)
Sepsis/acute pancreatitis	77 (11.5)	25 (13.6)	16 (11.2)
Cardiac failure/fluid overload	29 (4.3)	9 (4.9)	11 (7.7)
Pneumonia	95 (14.2)	25 (13.6)	29 (20.3)
Stroke or coma	107 (16.0)	38 (20.7)	17 (11.9)
Aspiration/inhalation	36 (5.4)	2 (1.1)	7 (4.9)
Trauma	117 (17,5)	9 (4.9)	9 (6.3)
Others	79 (11.8)	11 (6.0)	16 (11.2)
SOFA	8.3 ± 3.1	10.4 ± 3.2	9.8 ± 3.4
VT, *ml/kg PBW*	6.9 ± 1.0	6.9 ± 1.0	6.8 ± 1.2
FiO_2	0.63 ± 0.21	0.63 ± 0.22	0.67 ± 0.21
Respiratory rate, *cycles/min*	19.9 ± 4.2	19.3 ± 4.1	20.9 ± 5.2
PEEP, *cmH_2O*	8.0 ± 2.9	7.5 ± 2.7	8.4 ± 2.9
Plateau pressure, *cmH_2O*	21.3 ± 4.7	23.8 ± 6.1	25.0 ± 5.7
Driving pressure, *cmH_2O*	13.3 ± 3.9	16.3 ± 5.7	16.5 ± 5.1
PaO_2, *mmHg*	99.8 ± 34.4	99.6 ± 36.1	97.5 ± 34.3
PaO_2/FiO_2, *mmHg*	170.3 ± 62.5	174.1 ± 68.6	162.4 ±69.3
$PaCO_2$, *mmHg*	45.9 ± 11.9	45.5 ± 12.2	47.5 ± 12.8
pH	7.33 ± 0.10	7.32 ± 0.12	7.30 ± 0.11
No. extrapulmonary OF	1.5 ± 1.0	2.1 ± 0.9	2.0 ± 1.1
Minute ventilation, L/min	8.8 ± 2.0	8.4 ± 2.0	8.8 ± 2.1

Values are reported as means ± standard deviation (SD). ***Abbreviations****: AHRF: acute hypoxemic respiratory failure, bpm: breaths per minute, d: days, ICU: intensive care unit, MV: mechanical ventilation, N/n/no: number of patients, OF: organ failure, PBW: predicted body weight, SOFA: sequential organ dysfunction assessment, T0: at the time of AHRF diagnosis.*

TABLE S9. Data at T48 of 996 patients with acute hypoxemic respiratory failure (AHRF) after AHRF diagnosis in relation to ICU outcome (survivor or dead) and duration of mechanical ventilation (2-7 days vs. >7 days).

**Variables**	**ICU survivor** (independent of MV duration)	**ICU death on MV 2-7 d**	**ICU death on MV>7**
N, n (%)	669 (67.2)	184 (18.5)	143 (14.4)
Age	60.2 ± 14.9	66.2 ± 12.9	65.7 ± 13.2
Sex, n (%)			
female	210 (31.4)	67 (36.4)	39 (27.3)
male	459 (68.6)	117 (63.6)	104 (72.7)
Comorbidities, n (%)			
Arterial hypertension	312 (46.6)	110 (59.8)	81 (56.6)
Diabetes	168 (25.1)	53 (28.8)	55 (38.5)
Obesity	154 (23.0)	40 (21.7)	32 (22.4)
COPD	102 (15.2)	30 (16.3)	30 (21.0)
Cardiac failure	89 (13.3)	32 (17.4)	30 (21.0)
Neoplastic diseases	74 (11.1)	30 (16.3)	32 (22.4)
Immunosuppressed	52 (7.8)	23 (12.5)	22 (15.4)
Chronic renal disease	43 (6.4)	16 (8.7)	21 (14.7)
Reasons for MV, n (%)			
Post-surgery	91 (13.6)	27 (14.7)	26 (18.2)
Cardiac arrest	38 (5.7)	38 (20.7)	12 (8.4)
Sepsis/acute pancreatitis	77 (11.5)	25 (13.6)	16 (11.2)
Cardiac failure/fluid overload	29 (4.3)	9 (4.9)	11 (7.7)
Pneumonia	95 (14.2)	25 (13.6)	29 (20.3)
Stroke or coma	107 (16.0)	38 (20.7)	17 (11.9)
Aspiration/inhalation	36 (5.4)	2 (1.1)	7 (4.9)
Trauma	117 (17.5)	9 (4.9)	9 (6.3)
Others	79 (11.8)	11 (6.0)	16 (11.2)
SOFA, *mean±SD*	6.8 ± 3.4	10.5 ± 4.0	9.6 ± 3.5
VT, *ml/kg PBW, mean±SD*	7.0 ± 1.0	6.9 ± 1.0	6.7 ± 1.2
FiO_2, *mean±SD*	0.49 ± 0.15	0.56 ± 0.20	0.56 ± 0.20
Respiratory rate, *cycles/min*	21.3 ± 4.6	22.2 ± 5.3	22.5 ± 5.0
PEEP, *cmH_2O, mean±SD*	8.5 ± 3.0	8.5 ± 3.5	8.9 ± 2.9
Pplat, *cmH_2O, mean±SD*	19.4 ± 4.2	23.6 ± 6.3	24.5 ± 5.5
DP, *cmH_2O, mean±SD*	10.8 ± 3.4	15.2 ± 5.4	15.6 ± 5.0
PaO_2, *mmHg, mean±SD*	109.7 ± 31.3	110.6 ± 50.0	109.8±32.2
PaO_2/FiO_2, *mmHg, mean±SD*	247.3 ± 78.2	225.1 ± 93.3	227.0±89.1
$PaCO_2$, *mmHg, mean±SD*	43.9 ± 7.7	46.1 ± 11.8	46.9 ± 10.4
pH, *mean±SD*	7.41 ± 0.06	7.35 ± 0.12	7.40 ± 0.08
No. extrapulmonary OF	1.3 ± 1.0	2.4 ± 1.1	2.2 ± 1.1
Minute ventilation, L/min	9.4 ± 2.1	9.5 ± 2.1	9.4 ± 2.2

Abbreviations: *AHRF: acute hypoxemic respiratory failure, COPD: chronic obstructive pulmonary disease, d: days, DP: driving pressure, ICU: intensive care unit, MV: mechanical ventilation, N/n/no: number of patients, OF: organ failure, PBW: predicted body weight, Pplat: plateau pressure, SD: standard deviation, SOFA: sequential organ dysfunction assessment, T0: at the time of AHRF diagnosis.*

Table S10. Comparison between ICU survivors with ICU death ventilated for 2-7 days and for >7 days, using three machine learning techniques [Multilayer Perceptron (MLP), Random Forest (RF), Support Vector Machine (SVM)] and multinomial regression analysis (MNR). We reported sensitivity, specificity, accuracy, positive predictive value (PPV), and negative predictive value (NPV).

Technique	Comparison of ICU survivors vs. ICU deaths on MV 2-7 days					Comparison of ICU survivors vs. ICU deaths on MV >7 days				
	Accuracy	Sensitivity	Specificity	PPV	NPV	Accuracy	Sensitivity	Specificity	PPV	NPV
MLP	0.86	0.296	0.994	0.93	0.87	0.872	0.373	0.968	0.80	0.889
RF	0.835	0.521	0.915	0.613	0.88	0.867	0.331	0.954	0.55	0.898
SVM	0.786	0.015	0.997	0.58	0.79	0.825	0.002	0.999	0.3	0.826
MNR	0.838	0.504	0.921	0.632	0.88	0.891	0.304	0.979	0.73	0.903

FIGURE S1. Timeline for the early prediction model in three categories: ICU survivor (independent of MV duration), ICU death on mechanical ventilation <7 days, and ICU death on mechanical ventilation >7 days, all in patients with acute hypoxemic respiratory failure (AHRF). At the patient's time of eligibility (i.e., when patients develop AHRF), the patient's risk of future outcome at ICU discharge (liberated from MV, extubated, or non-survivor) was predicted in patients using the first 48 hours of data.

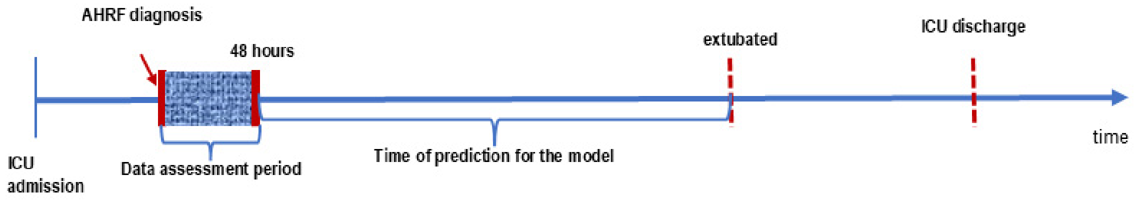

FIGURE S2. Receiver operating characteristic curves of assigned groups (ICU deaths on MV≤7 days or ICU deaths on MV>7 days) vs. ICU survivors in 996 patients with acute hypoxemic respiratory failure in a multinomial regression model. *Abbreviations: AUC: area under the receiver characteristic curve; CI: confidence interval; MV: mechanical ventilation; N: number of patients; NPV: negative predictive value; PPV: positive predictive value.*

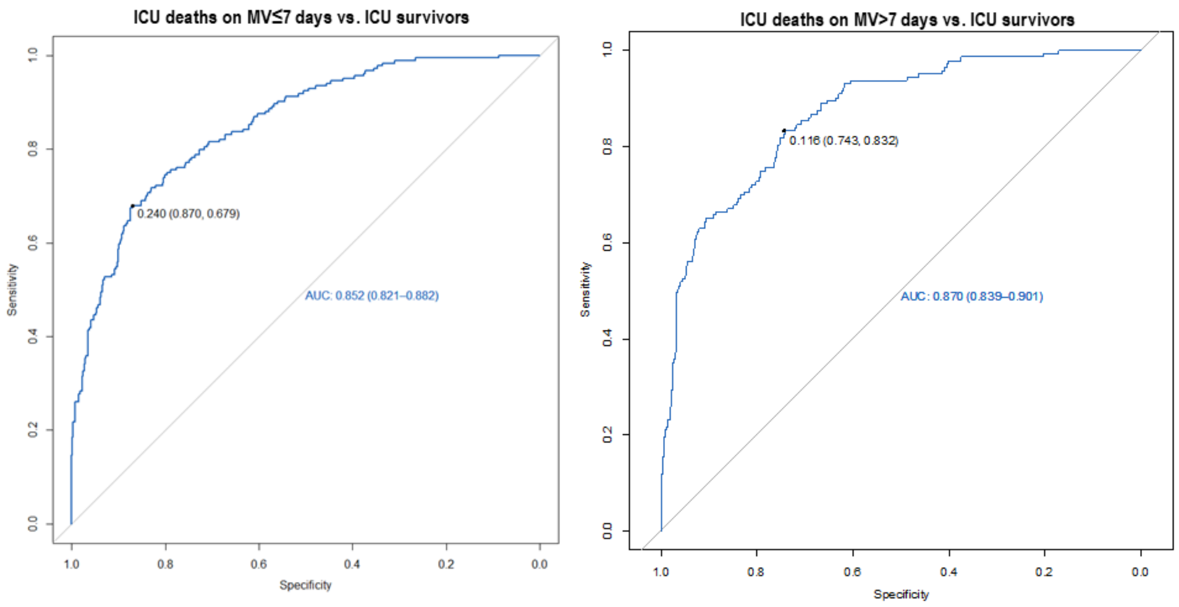

Groups	N	AUC (95%CI)	Sensitivity	Specificity	Accuracy	PPV	NPV
ICU deaths on MV≤7 days vs. survivors	853 = 669 + 184	0.852 (0.821-0.882)	0.70	0.87	0.83	0.59	0.91
ICU deaths on MV>7 days vs. survivors	812 = 669 + 143	0.870 (0.839-0.901)	0.83	0.74	0.76	0.41	0.95

Figure S3. Calibration plots of the Multilayer Perceptron-based prediction model, comparing ICU survivors vs. ICU deaths on mechanical ventilation for 2-7 days (A) and ICU deaths on mechanical ventilation for more than 7 days (B). The intercept relates to calibration-in-large, which compares mean observed predicted risks. The calibration slope reflects the coefficient of the calibration plot. The *c*-statistic indicates the discriminative ability.

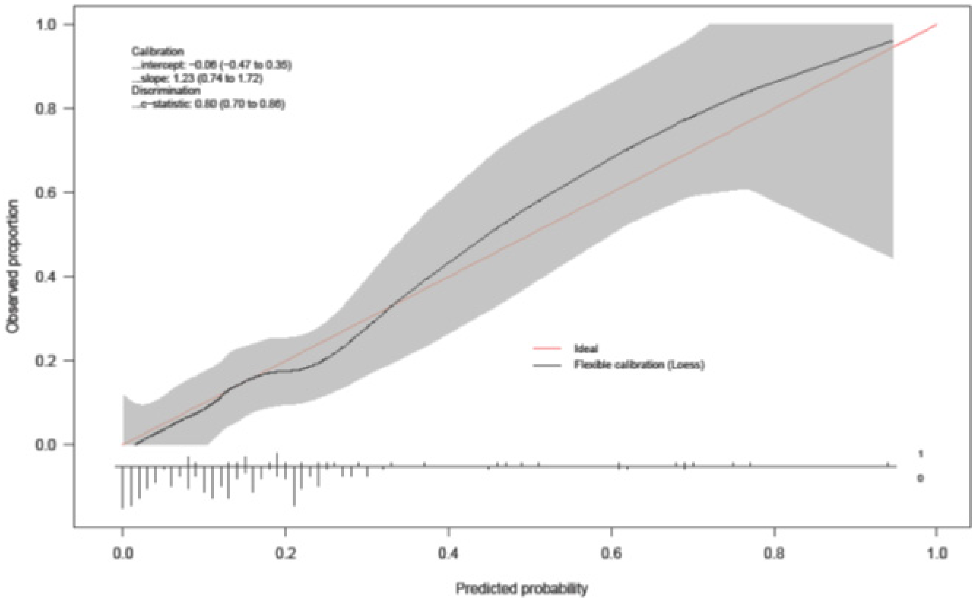

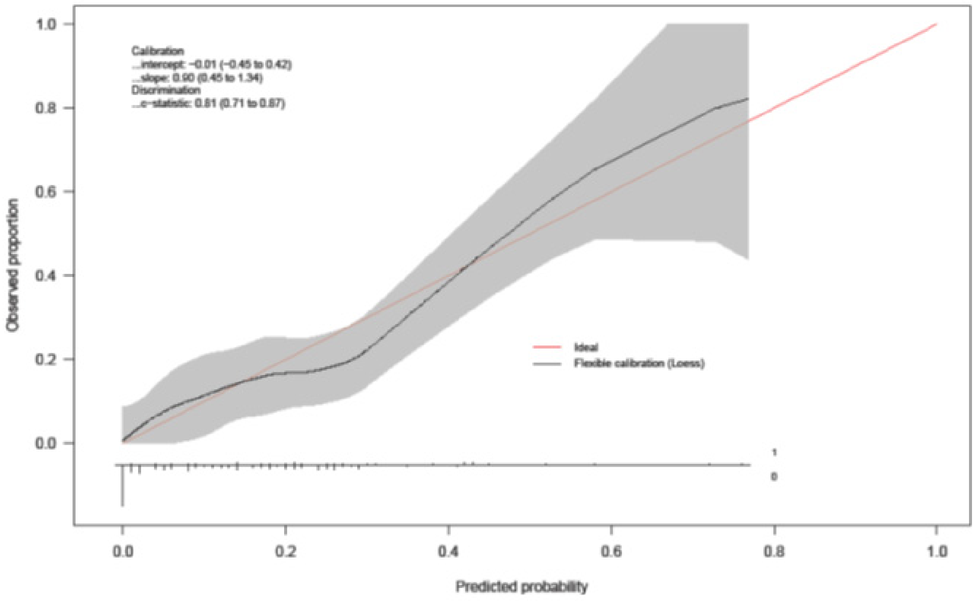

SUPPLEMENTARY REFERENCES

1. Villar J, Mora-Ordoñez JM, Soler JA, Mosteiro F, Vidal A, Ambrós A, Fernández L, Murcia I, Civantos B, Romera MA, et al. The PANDORA study: prevalence and outcome of acute hypoxemic respiratory failure in the pre-Covid-19 era. Crit Care Explor 2022; 4:e0684.
2. Parsa-Parsi RW. The International Code of Medical Ethics of the World Medical Association. JAMA 2022; 328:2018-2021.
3. Collins GS, Reitsma JB, Altman DG, Moons KG. Transparent reporting of a multivariable prediction model for individual prognosis or diagnosis (TRIPOD): The TRIPOD statement. BMJ 2015; 350:g7594.
4. Leisman DE, Harhay MO, Lederer DJ, Abramson M, Adjei AA, Bakker J, Ballas ZK, Barreiro E, Bell SC, Bellomo R, et al. Development and reporting of prediction models: Guidance for authors from editors of respiratory, sleep, and critical care journals. Crit Care Med 2020; 48:623-633.
5. Villar J, González-Martín JM, Fernández C, Soler JA, Ambrós A, Pita-García L, Fernández L, Ferrando C, Arocas B, González-Vaquero M, The PredictION Of Duration Of mEchanical vEntilation In Ards Pioneer Network. Predicting the length of mechanical ventilation in acute respiratory distress syndrome using machine learning: The PIONEER Study. J Clin Med 2024; 13:1811.
6. Calster BV, Vergouwe Y, Looman CWN, Belle VV, Timmerman D, Steyerberg EW. Assessing the discriminative ability of risk models for more than two outcome categories. Eur J Epidemiol 2012; 27:761-770.
7. Steyerberg EW, Harrel FE: Prediction models need appropriate internal, internal-external, and external validation. J Clin Epidemiol 2016; 69:245-247.
8. Steyerberg EW, Harrel FE, Borsboom GJJM, Eijkemans MJCR, Vergouwe Y, Habbema JDF: Internal validation of predictive models: efficiency of some procedures for logistic regression analysis. J Clin Epidemiol 2011; 54:774-781.
9. Villar J, Martínez D, Mosteiro F, Ambrós A, Añón JM, Ferrando C, Soler JA, Montiel R, Vidal A, Conesa-Cayuela LA, Blanco J; Stratification and outcome of acute respiratory distress syndrome (STANDARDS) network. Is overall mortality the right composite endpoint in clinical trials of acute respiratory distress syndrome? Crit Care Med 2018; 46:892-899.

10. Acute Respiratory Distress Syndrome Network. Ventilation with lower tidal volumes as compared with traditional tidal volumes for acute lung injury and the acute respiratory distress syndrome. N Engl J Med 2000; 342:1301-1308.
11. Kacmarek RM. Noninvasive respiratory support for postextubation respiratory failure. Respir Care 2019; 64:658-678.
12. Kacmarek RM, Villar J, Sulemanji D, Montiel R, Ferrando C, Blanco J, et al. Open lung approach for the acute respiratory distress syndrome: A pilot, randomized controlled trial. Crit Care Med 2016; 44:32-42.
13. Knaus WA, Draper EA, Wagner DP, Zimmerman JE. APACHE II: a severity of disease classification system. Crit Care Med 1985; 13:818-829.
14. Vincent JL, de Mendonça A, Cantraine F, Moreno R, Takala J, Suter PM, Sprung CL, Colardyn F, Blecher S. Use of the SOFA score to assess the incidence of organ dysfunction/failure in intensive care units: results of a multicenter, prospective study. Working group on "sepsis-related problems" of the European Society of Intensive Care Medicine. Crit Care Med 1998; 26:1793-1800.
15. Singer M, Deutschman CS, Seymour CW, Shankar-Hari M, Annane D, Bauer M, Bellomo R, Bernard GR, Chiche JD, Coopersmith CM, et al. The third international consensus definitions for sepsis and septic shock (sepsis-3). JAMA 2016; 315:801-810.
16. Eke G, Bloos F, Wilson DC, Meybohm P, SepNet Critical Care Trials Group. Identification of developing multiple organ failure in sepsis patients with low or moderate SOFA scores. Crit Care 2018; 22:147.
17. Ranieri VM, Rubenfeld GD, Thompsom BT, Ferguson ND, Caldwell E, Fan E, Camporota L, Slutsky AS. Acute respiratory distress syndrome. The Berlin definition. JAMA 2012; 307:2526-2533.
18. Rauf A, Sachdev A, Venkataraman ST, Dinand V. Dynamic airway driving pressure and outcomes in children with acute hypoxemic respiratory failure. Respir Care 2021; 66:403-409.
19. Gutierrez G. Artificial intelligence in the intensive care unit. Crit Care 2020; 24:101.
20. Vrieze SI. Model selection and psychological theory: a discussion of the differences between the Akaike information criterion (AIC) and the Bayesian information criterion (BIC). Psychol Methods 2012; 17:228-243.
21. Mandrekar JN. Receiver operating characteristic curve in diagnostic test assessment. J Thorac Oncol 2010; 5:1315-1316.

22. Rashid M, Ramakrishnan M, Pulikkel V, Nandish S, Nair S, Shanbhag V, Thunga G. Artificial intelligence in acute respiratory distress syndrome: a systematic review. Artif Intell Med 2022; 131:102361.
23. Sayed M, Riaño D, Villar J. Predicting duration of mechanical ventilation in acute respiratory distress syndrome using supervised machine learning. J Clin Med 2021; 10:3824.
24. Boulesteix AL, Janitza S, Kruppa J, König IR. Overview of random forest methodology and practical guidance with emphasis on computational biology and bioinformatics. 2012; 2:493–507 (free access online https://wires.onlinelibrary.wiley.com/doi/abs/10.1002/widm.1072
25. Khalilzad Z, Hasasneh A, Tadj C. Newborn cry-based diagnostic system to distinguish between sepsis and respiratory distress syndrome using combined acoustic features. Diagnostics (Basel) 2022; 12:2802.
26. Jeon ET, Lee HJ, Park TY, Jin KN, Ryu B, Lee HW, Kim DH. Machine learning-based prediction of in-ICU mortality in pneumonia patients. Sci Rep 2023; 13:11527.
27. Saxena A, Mathur N, Pathak P, Tiwari P, Mathur SK. Machine learning model based on insulin resistance metagenes underpins genetic basis of type 2 diabetes. Biomolecules 2023; 13:432.
28. Ioannidis JPA. The proposal to lower P value thresholds to 0.005. JAMA 2018; 319:1429-1430.
29. Linck EJG, Goliher EC, Semler NW, Chupak MM. Toward precision in critical care research: methods for observational and interventional studies. Crit Care Med 2024; 52:1439-1450.
30. Maslove DM, Tang B, Shankar-Hari M, Lawler PR, Angus DC, Baillie JK, Baron RM, Bauer M, Buchmann TG, Calfee CS, et al. Redefining critical illness. Nat Med 2022; 28:1141-1148.
31. Rubolotta F, Bahrami S, Marshall DC, Komorouski M. Machine learning tools for acute respiratory distress syndrome detection and prediction. Crit Care Med 2024; 52:1768-1780.
32. Taran S, Liu K, McCredie VA, Peñuelas O, Burns KEA, Frutos-Vivar F, Scales DC, Ferguson ND, Singh JM, Malhotra AK, et al. Decisions to withdraw or withhold life-sustaining therapies in patients with and without acute brain injury: a secondary analysis of two prospective cohort studies. Lancet Respir Med 2025; 13:338-347.
33. Taylor SP, Eaton T, Rios A, Boyd D, Tapp H, McWilliams A, Chon SH, Halpern S, Angus DC, McCurly L, et al. Proactive telehealth-based sepsis transition and recovery support, hospital readmission, and mortality. A randomized clinical trial. JAMA Intern Med 2025; 185:1238-1246.

34. Burns KEA, Raptis S, Nisenbaum R, Rizvi L, Jones A, Bakshi J, Tan W, Meret A, Cook DJ, Lellouche et al. International practice variation in weaning critically ill adults from invasive mechanical ventilation. Ann Am Thorac Soc 2018; 15:494-502.
35. Burns KEA, Allan JE, Lee E, Santos-Taylor M, Kay P, Greco P, Every H, Mooney O, Tanios M, Tan E, et al. Liberation from mechanical ventilation using Extubation Advisor Decision Support (LEADS): protocol for a multicenter pilot trial. BMJ Open 2025; 15:e093853.

APPENDIX S1. List of centers and investigators involved in the PREMIER study.

Centers	Department/Hospital	Address and city/province	Investigators
1	Intensive Care Unit,<br>Hospital Universitario de La Paz	Paseo de la Castellana 261<br>28046 Madrid, Spain	José M. Añón<br>Belén Civantos<br>Mónica Hernández
2	Intensive Care Unit,<br>Hospital Virgen de La Luz	Hermandad Donantes de Sangre<br>1 16002 Cuenca, Spain	Elena González<br>Rosario Solano
3	Intensive Care Unit,<br>Complejo Asistencial Universitario de León	Altos de Nava s/n<br>24001 León, Spain	Ana M. Domínguez-Berrot<br>F. Javier Díaz-Domínguez<br>Raúl I. González Luengo<br>Myriam González-Vaquero
4	Post-Surgical Care Unit,<br>Hospital Clínico Universitario de Valencia	Blasco Ibáñez 17<br>46010 Valencia, Spain	Carlos Ferrando*<br>Blanca Arocas<br>Marina Soro<br>Andrea Gutiérrez<br>Gerardo Aguilar
5	Intensive Care Unit,<br>Hospital Universitario Río Hortega	Dulzaina 2<br>47012 Valladolid, Spain	Lorena Fernández<br>Jesús Sánchez-Ballesteros<br>Arturo Muriel<br>Pablo Blanco-Schweizer<br>José Ángel de Ayala<br>Jesús Blanco
6	Intensive Care Unit,<br>Hospital Clínico Universitario de Valladolid	Avda. Ramón y Cajal 3<br>47003 Valladolid, Spain	David Andaluz-Ojeda**<br>Leonor Nogales<br>Laura Parra
7	Intensive Care Unit,<br>Hospital Universitario Virgen de Arrixaca	Ctra. Madrid-Cartagena s/n<br>30120 El Palmar, Murcia, Spain	Juan A. Soler<br>Domingo Martínez<br>Ana M. del Saz-Ortiz<br>Luís A. Conesa-Cayuela
8	Intensive Care Unit,<br>Hospital General Universitario de Ciudad Real	Obispo Rafael Torija s/n<br>13005 Ciudad Real, Spain	Alfonso Ambrós<br>Ramón Ortiz-Díaz-Miguel<br>Rafael del Campo<br>Carmen Martínez-Rodríguez<br>Ana Bueno-González<br>Carmen Hornos-López
9	Intensive Care Unit,<br>Hospital Universitario NS de Candelaria	Ctra. Del Rosario 145<br>38010 Santa Cruz de Tenerife, Spain	Raquel Montiel<br>Dácil Parrilla<br>Eduardo Peinado<br>Lina Pérez-Méndez

10	Intensive Care Unit,<br>Hospital Universitario 12 de Octubre	Avda. de Córdoba s/n<br>28041 Madrid, Spain	Isidro Prieto<br>Mario Chico
11	Intensive Care Unit,<br>Hospital Universitario Puerta de Hierro	Manuel de Falla 1<br>28222 Majadahonda, Madrid, Spain	Miguel A. Romera<br>Ana Amaro-Harpigny<br>Carlos Chamorro-Jambrina
12	Intensive Care Unit,<br>Hospital Universitario Regional	*Carlos Haya* s/n<br>29010 *Málaga, Spain*	Juan M. Mora-Ordoñez<br>J. F. Martínez-Carmona<br>Álvaro Valverde-Montoro<br>Victoria Olea-Jiménez
13	Intensive Care Unit,<br>Hospital NS del Prado	Ctra Madrid Km 114, 45600<br>Talavera de la Reina, Toledo, Spain	Paco Alba<br>Ruth Corpas
14	Intensive Care Unit,<br>Hospital Universitario de A Coruña	As Xubias 84<br>15006 A Coruña, Spain	Fernando Mosteiro<br>Marta Rey-Abalo<br>Lidia Pita-García<br>Ana M. Díaz-Lamas
15	Intensive Care Unit,<br>Hospital El Bierzo, Ponferrada	Médicos sin Fronteras 7<br>24404 Ponferrada, León, Spain	Eleuterio Merayo<br>Chanel Martínez<br>Ángeles de Célis-Álvarez
16	Intensive Care Unit,<br>Hospital La Mancha Centro	Avda. Constitución 3, 13600<br>Alcázar de S. Juan, Ciudad Real, Spain	Carmen Martín-Delgado
17	Post-operative Care Unit,<br>Hospital Universitario Río Hortega	Dulzaina 2<br>47012 Valladolid, Spain	César Aldecoa<br>Alba Pérez<br>Jesús Rico-Feijoo
18	Post-operative Care Unit,<br>Hospital Universitrario Ramón y Cajal	Ctra. Colmenar Viejo Km 9.1<br>28034 Madrid, Spain	David Pestaña<br>Adrián Mira<br>Pilar Cobeta
19	Intensive Care Unit,<br>Hospital Universitario Mutua Terrassa	Plaça del Dr. Robert 5<br>08221 Terrassa, Barcelona, Spain	María del Mar Fernández
20	Intensive Care Unit,<br>Hospital Virgen de la Concha	Avda. Requejo 35<br>49022 Zamora, Spain	Concepción Tarancón<br>Silvia Cortés-Díaz
21	Intensive Care Unit,<br>Hospital Fundación Jiménez Díaz	Avda. Reyes Católicos 2<br>28040 Madrid, Spain	Anxela Vidal<br>Denis Robaglia<br>César Pérez
22	Intensive Care Unit,<br>Hospital Universitario de Albacete	Hermanos Falcó 37<br>02006 Albacete, Spain	Isabel Murcia<br>Ángel E. Pereyra-Pache

23	Research Unit,<br>Hospital Universitario Dr. Negrín	Barranco de la Ballena s/n.<br>35019 Las Palmas de Gran Canaria, Spain	Jesús Villar<br>Jesús M. González-Martín<br>Cristina Fernández<br>Estrella Gómez-Bentolila<br>(not enrolling patients)
24	Department of Biomedical Data Sciences, Leiden University Meical Center	Albinusdreef 2, 2333 ZA Leiden, The Netherlands	Ewout W. Steyerberg<br>(not enrolling patients)
25	Department of Adult Critical Care, Guy's and St. Thomas' NHS Foundation Trust	Westminster Bridge Road, London, SE1 7EH, U.K.	Luigi Camporota<br>(not enrolling patients)
26	Department of Anaesthesia, Intensive Care and Pain Medicine.	Cardiff University<br>Cardiff CF14 4XN,<br>United Kingdom	Tamas Szakmany<br>(not enrolling patients)

NOTE: One center (unlisted) abandoned the study during the first 2-month period because of absence of its local investigator.

(*) current affiliation: Department of Anesthesia and Critical Care, Hospital Clinic, Barcelona, Spain.

(**) current affiliation: Intensive Care Unit, Complejo Asistencial Universitario de Palencia, Palencia, Spain.
